# DNA methylation analysis with methylation‐sensitive high‐resolution melting (MS‐HRM) reveals gene panel for glioma characteristics

**DOI:** 10.1111/cns.13443

**Published:** 2020-08-11

**Authors:** Aleksandra Majchrzak‐Celińska, Emilia Dybska, Anna‐Maria Barciszewska

**Affiliations:** ^1^ Department of Pharmaceutical Biochemistry Poznan University of Medical Sciences Poznań Poland; ^2^ Department of Pediatric Gastroenterology and Metabolic Diseases Poznan University of Medical Sciences Poznań Poland; ^3^ Intraoperative Imaging Unit Chair and Department of Neurosurgery and Neurotraumatology Poznan University of Medical Sciences Poznań Poland; ^4^ Department of Neurosurgery and Neurotraumatology Heliodor Swiecicki Clinical Hospital Poznań Poland

**Keywords:** biomarker, DNA methylation, gliomas, MS‐HRM

## Abstract

**Introduction:**

Local DNA hypermethylation is a potential source of cancer biomarkers. While the evaluation of single gene methylation has limited value, their selected panel may provide better information.

**Aims:**

This study aimed to analyze the promoter methylation level in a 7‐gene panel in brain tumors and verifies the usefulness of methylation‐sensitive high‐resolution melting (MS‐HRM) for this purpose.

**Methods:**

Forty‐six glioma samples and one non‐neoplastic brain sample were analyzed by MS‐HRM in terms of *SFRP1*, *SFRP2*, *RUNX3*, *CBLN4*, *INA*, *MGMT*, and *RASSF1A* promoter methylation. The results were correlated with patients’ clinicopathological features.

**Results:**

DNA methylation level of all analyzed genes was significantly higher in brain tumor samples as compared to non‐neoplastic brain and commercial, unmethylated DNA control. *RASSF1A* was the most frequently methylated gene, with statistically significant differences depending on the tumor WHO grade. Higher *MGMT* methylation levels were observed in females, whereas the levels of *SFRP1* and *INA* promoter methylation significantly increased with patients’ age. A positive correlation of promoter methylation levels was observed between pairs of genes, for example, *CBLN4* and *INA* or *MGMT* and *RASSF1A*.

**Conclusions:**

Our 7‐gene panel of promoter methylation can be helpful in brain tumor diagnosis or characterization, and MS‐HRM is a suitable method for its analysis.

## Graphical abstract

Promoter methylation of a 7‐gene panel can be helpful in brain tumor diagnosis or characterization, and MS‐HRM is a suitable method for its analysis.

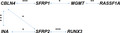

## 1 INTRODUCTION

Aberrant DNA methylation is one of the hallmarks of cancer. Hypermethylation of tumor suppressor gene promoter sequences, as well as global DNA hypomethylation, characterizes all tumors, including CNS cancers. Distinctive epigenetic changes alter gene expression, contributing to disease pathogenesis, but can also be helpful in diagnosis and prediction of prognosis or treatment response. The updated 2016 World Health Organization (WHO) classification of CNS tumors indicated that beyond clinical and symptom‐based examinations, also molecular characterization analysis should be applied.^1^ It did not include epigenetic tumor characteristics yet, but recent data presented by Capper et al show that classification of CNS tumors based on DNA methylation profiling results in the revision of the initial histopathological diagnosis in 12% of cases (of 2,801 reference samples analyzed).^2^ Moreover, DNA methylation analysis can also serve as a prognostic indicator. In this context, G‐CIMP (Glioma CpG island methylator phenotype)–positive phenotype correlates with the presence of *IDH* mutation and is associated with a good prognosis in GBM (glioblastoma multiforme).^3^ Eventually, methylation biomarkers can also predict the response to specific therapy and guide patients’ treatment. Multiple large‐scale clinical studies proved that GBM patients with O^6^‐methylguanine‐DNA methyltransferase (*MGMT)* promoter hypermethylation benefit from alkylating agent therapy, especially temozolomide (TMZ).^4^, ^5^, ^6^, ^7^ Thus, *MGMT* is now the most frequently used methylation‐associated biomarker in neuro‐oncology. However, to date, there is no consensus on the optimal method for the detection of *MGMT* as well as other DNA methylation biomarkers in the clinical setting.^8^

There is a variety of analytical methods for DNA methylation analysis, allowing both whole‐genome methylation profiling and *locus*‐specific DNA methylation assays. The first approach mentioned is often used in the discovery phase allowing the identification of differentially methylated regions and their relevance to the disease. On the other hand, the second approach—*locus*‐specific DNA methylation analysis, is an optimal technique in the clinics, since most of the established biomarkers rely on DNA methylation differences in the limited number of CpG dinucleotides.^9^ Overall, the optimal DNA methylation detection method should be sensitive, specific, quick, cost‐effective, and suitable for screening of large sets of clinical samples.^10^ Methylation‐sensitive high‐resolution melting (MS‐HRM) has all of these features.^11^, ^12^, ^13^

In this method, establishing the DNA methylation level of a particular sequence is based on the differences in the melting profiles of its PCR amplicon. Bisulfite treatment, preceding PCR amplification, creates the difference in the nucleotide sequence corresponding to the presence or absence of methyl groups. In this regard, unmethylated cytosine is oxidatively deaminated to uracyl (read as thymine during PCR), whereas methylated cytosine remains cytosine. Cytosine‐guanine pair melts in higher temperatures, as compared to the adenine‐thymine pair, resulting in markedly different melting profiles. Due to its simplicity and high reproducibility, MS‐HRM is now gaining importance both in screening and in determining new molecular biomarkers.^13^, ^14^

The DNA methylation studies in CNS neoplasms usually concentrate on single genes^15^ or evaluate methylomes or methylation profiles using microarrays.^16^ The first approach carries a disadvantage, as cancer is not a single gene disease.^17^ The second methodology provides an enormous amount of data that create a complex picture of a disease, but fail to serve as a simple test.^2^, ^18^, ^19^, ^20^ The golden mean would be defining a set of genes crucial for diagnostic and therapeutic purposes. Thus, the aim of this study was to explore the biomarker potential of the promoter methylation level of a 7‐gene panel that we propose for CNS cancer detection and characterization. In this regard, the obtained data were correlated with patients’ clinicopathological features such as gender, age, histological tumor type, and WHO grade, as well as overall survival time (OS). The idea behind the gene set was to represent the crucial processes disturbed in gliomagenesis, that is, signal transduction (Wnt/β‐catenin pathway) or neuronal development and organization. In the panel, we included *SFRP1*, *SFRP2*, *RUNX3*, *CBLN4*, *INA*, *MGMT*, and *RASSF1A*. The choice of genes was determined based on the established or potential role of promoter methylation of these genes in glioma detection and patients' management. *SFRP1*, *SFRP2*, and *RUNX3* are negative regulators of the Wnt/β‐catenin pathway. Secreted frizzled‐related protein (SFRP) genes, *SFRP1* and *SFRP2*, belong to the secreted glycoprotein family. Epigenetic silencing of *SFRP1* may cause dysregulation of cell proliferation, migration, and invasion, which leads to cancer cell formation, disease progression, poor prognosis, and treatment resistance.^21^ SFRP2 is regarded as the most potent antagonist of Wnt signaling. However, SFRP2 can also exert an angiogenic effect in renal and lung cancer.^22^ Runt‐related transcription factor functions as a tumor suppressor, and the gene (*RUNX3*) is frequently deleted or transcriptionally silenced in cancer.^23^, ^24^ Its inactivation is frequently caused by promoter methylation.^21^, ^25^, ^26^ Previously, we found that *RUNX3* methylation correlates with WHO tumor grade and glioma patients’ age.^27^, ^28^ Moreover, *SFRP1* methylation predicts shorter survival of patients with gliomas.^26^ INA (internexin neuronal intermediate filament protein α) is a major component of the intermediate filament network in the cytoplasm of small interneurons and cerebellar granule cells and plays a role in neuronal development.^29^ It is implicated to contribute to neurodegenerative disorders.^29^ INA is overexpressed mostly in oligodendroglial phenotype gliomas and is related to 1p/19q codeletion with >70% specificity. Therefore, it is a favorable prognostic marker.^30^ Recently, a set of CpG *loci* differentially hypermethylated in GBM short‐term survivors (overall survival < 1 year) vs. long‐term survivors (overall survival > 3 years) was identified.^31^ According to this report, methylation of *INA* was one of the top hypermethylated *loci* and indicated short‐term survival. Another differentially hypermethylated gene was *CBLN4* (cerebellin 4 precursor), a *trans*‐synaptic cell adhesion molecule, which is important for the synaptic organization of specific subsets of neurons^32^ and it was not previously linked to brain tumors or other cancers.^6^ *MGMT* is already established as a predictive factor in patients with GBM treated with temozolomide (TMZ).^4^, ^33^ It encodes O^6^‐methylguanine‐DNA methyltransferase, which acts by removing alkyl adducts from the O^6^ position of guanine at DNA level, thus antagonizing the cytotoxic effects of alkylating agents, including TMZ, gold standard anti‐GBM therapeutic.^7^ Recently, also the prognostic value of that marker was established, making it a great descriptor of a link between disease character and therapy response.^16^ Loss or altered expression of *RASSF1A*, Ras association domain family 1 isoform A encoding gene, has been associated with the pathogenesis of a variety of cancers.^34^ In our previous study, hypermethylation of *RASSF1A* analyzed in circulating tumor‐derived DNA differentiated primary from metastatic brain cancers.^35^ The summary of the proposed panel characteristics is presented in Table S1.

## 2 METHODS

### 2.1 Sample characteristics

This study was performed on brain glioma samples obtained from 46 patients during surgery at the Department of Neurosurgery and Neurotraumatology, Poznan University of Medical Sciences, and on one non‐neoplastic brain tissue. Patients’ age ranged from 16 to 83 years, whereas the largest subgroup consisted of patients within the age range of 51‐60 years (18 subjects). The median age of patients at the time of tumor diagnosis was 50.8 ± 15.5 years. In the group, there were 27 (58.7%) males and 19 (41.3%) females. The most abundant histological subgroups were as follows: anaplastic astrocytomas (13 patients—28.3%, 2 of them with recurrent tumors), and glioblastomas (19 patients—41.3%, 7 of them with recurrent tumors). Single cases represented other histological types. Twenty patients were described as WHO grade IV, 19—grade III, 5—grade II, and 2—grade I. The histological types and grades (from I—highly differentiated, least malignant to IV—low differentiated, most malignant), age, and gender are shown in Table 1.

**TABLE 1:** The list of patients with brain gliomas evaluated in the present study with their basic characteristics (histopathological diagnosis, WHO grade, gender, age, and overall survival time), as well as promoter DNA methylation of analyzed genes (*SFRP1*, *SFRP2*, *RUNX3*, *CBLN4*, *INA*, *MGMT*, *RASSF1A*)

Indication							
Methylation level [%]	0	>0, ≤5	>5, ≤10	>10, ≤25	>25, ≤50	>50, ≤75	>75, ≤100

No	Histopathological classification	WHO grade	Gender	Age [years]	Overall survival [months]	*SFRP1*	*SFRP2*	*RUNX3*	*CBLN4*	*INA*	*MGMT*	*RASSF1A*
1	*GBM*	IV	M	60	8	>10, ≤25%	>0, ≤5%	>0, ≤5%	>0, ≤5%	>0, ≤5%	0%	>10, ≤25%
2	*GBM*	IV	M	58	17	0%	0%	0%	0%	0%	0%	0%
3	*GBM*	IV	M	58	10	>0, ≤5%	>0, ≤5%	0%	0%	0%	0%	0%
4	*GBM*	IV	M	58	9	>10, ≤25%	>0, ≤5%	>0, ≤5%	>0, ≤5%	0%	0%	>10, ≤25%
5	*GBM*	IV	M	56	nk	>10, ≤25%	0%	0%	>0, ≤5%	>5, ≤10%	0%	>10, ≤25%
6	*GBM*	IV	M	54	16	>25, ≤50%	>0, ≤5%	0%	>0, ≤5%	0%	0%	0%
7	*GBM*	IV	M	47	10	0%	>0, ≤5%	>0, ≤5%	0%	0%	0%	>0, ≤5%
8	*GBM*	IV	M	45	21	0%	nk	0%	0%	0%	0%	0%
9	*GBM*	IV	M	41	nk	>25, ≤50%	0%	0%	>0, ≤5%	0%	0%	0%
10	*GBM*	IV	F	70	3	>25, ≤50%	0%	0%	0%	0%	>25, ≤50%	0%
11	*GBM*	IV	F	65	nk	>10, ≤25%	0%	0%	0%	>0, ≤5%	0%	>25, ≤50%
12	*GBM*	IV	F	60	36	>25, ≤50%	0%	0%	>10, ≤25%	>0, ≤5%	>10, ≤25%	>25, ≤50%
13	*GBM*	IV	F	52	nk	>0, ≤5%	>0, ≤5%	0%	0%	0%	>10, ≤25%	0%
14	*GBM recidivans*	IV	M	31	8	0%	0%	0%	0%	0%	0%	0%
15	*GBM recidivans*	IV	M	31	8	0%	0%	0%	0%	0%	0%	0%
16	*GBM recidivans*	IV	F	64	nk	>10, ≤25%	0%	>10, ≤25%	0%	0%	0%	0%
17	*GBM recidivans*	IV	F	60	nk	0%	0%	0%	0%	0%	>0, ≤5%	>0, ≤5%
18	*GBM recidivans*	IV	F	59	4	0%	>0, ≤5%	0%	0%	>0, ≤5%	0%	0%
19	*GBM recidivans*	IV	F	50	nk	>10, ≤25%	0%	0%	0%	0%	>5, ≤10%	0%
20	*Gliosarcoma*	IV	F	75	10	>10, ≤25%	>5, ≤10%	>25, ≤50%	>0, ≤5%	>0, ≤5%	>10, ≤25%	>0, ≤5%
21	*Anaplastic astrocytoma*	III	M	83	nk	>25, ≤50%	>0, ≤5%	0%	>0, ≤5%	>0, ≤5%	>10, ≤25%	>10, ≤25%
22	*Anaplastic astrocytoma*	III	M	60	10	0%	>0, ≤5%	0%	0%	>0, ≤5%	>25, ≤50%	>10, ≤25%
23	*Anaplastic astrocytoma*	III	M	59	nk	>10, ≤25%	>0, ≤5%	>5, ≤10%	>0, ≤5%	>0, ≤5%	0%	0%
24	*Anaplastic astrocytoma*	III	M	58	11	0%	0%	>5, ≤10%	0%	0%	0%	>0, ≤5%
25	*Anaplastic astrocytoma*	III	M	45	nk	>5, ≤10%	>0, ≤5%	>5, ≤10%	>0, ≤5%	>0, ≤5%	0%	>10, ≤25%
26	*Anaplastic astrocytoma*	III	M	29	18	>25, ≤50%	>0, ≤5%	>0, ≤5%	>0, ≤5%	0%	0%	>5, ≤10%
27	*Anaplastic astrocytoma*	III	F	73	nk	>25, ≤50%	>5, ≤10%	>10, ≤25%	>0, ≤5%	>10, ≤25%	>50, ≤75%	>0, ≤5%
28	*Anaplastic astrocytoma*	III	F	66	nk	>10, ≤25%	>0, ≤5%	>5, ≤10%	0%	>0, ≤5%	0%	0%
29	*Anaplastic astrocytoma*	III	F	48	nk	0%	>0, ≤5%	>0, ≤5%	>0, ≤5%	>0, ≤5%	0%	0%
30	*Anaplastic astrocytoma*	III	F	48	nk	0%	0%	0%	>0, ≤5%	>0, ≤5%	>0, ≤5%	0%
31	*Anaplastic astrocytoma*	III	F	41	42	0%	0%	0%	0%	0%	>10, ≤25%	>50, ≤75%
32	*Anaplastic astrocytoma recidivans*	III	M	53	nk	0%	>0, ≤5%	0%	0%	0%	0%	>25, ≤50%
33	*Anaplastic astrocytoma recidivans*	III	F	47	8.5	>25, ≤50%	0%	0%	>0, ≤5%	0%	>10, ≤25%	>50, ≤75%
34	*Astrocytoma gemistocyticum in astrocytoma angioplasticum vertens*	III	F	28	nk	0%	0%	0%	0%	0%	>10, ≤25%	>25, ≤50%
35	*Glioma anaplasticum NOS*	III	M	58	23	0%	0%	0%	0%	0%	>10, ≤25%	>25, ≤50%
36	*Glioma anaplasticum recidivans*	III	M	59	3	0%	0%	0%	0%	0%	0%	0%
37	*Oligoastrocytoma anaplasticum*	III	M	29	23	>25, ≤50%	0%	0%	0%	0%	>10, ≤25%	>50, ≤75%
38	*Oligoastrocytoma anaplasticum*	III	F	77	3.5	>25, ≤50%	0%	0%	>0, ≤5%	>0, ≤5%	0%	>25, ≤50%
39	*Clear cell ependymoma*	III	F	19	3	0%	>0, ≤5%	0%	0%	0%	0%	>25, ≤50%
40	*Astrocytoma fibrillare partim protoplasmaticum*	II	M	51	12.5	>10, ≤25%	>0, ≤5%	0%	>0, ≤5%	0%	>10, ≤25%	>50, ≤75%
41	*Astrocytoma fibrillare partim gemistocyticum*	II	M	37	nk	>10, ≤25%	0%	0%	0%	0%	>0, ≤5%	>50, ≤75%
42	*Astrocytoma fibrillare*	II	F	38	18	>10, ≤25%	0%	>0, ≤5%	nk	nk	>0, ≤5%	>10, ≤25%
43	*Oligodendroglioma isomorphum*	II	M	59	6	>25, ≤50%	>0, ≤5%	>5, ≤10%	0%	0%	>10, ≤25%	>50, ≤75%
44	*Ganglioglioma*	II	M	37	nk	>25, ≤50%	nk	>0, ≤5%	0%	0%	>10, ≤25%	>25, ≤50%
45	*Subependymal giant cell astrocytoma*	I	M	23	24	0%	0%	0%	0%	0%	0%	0%
46	*Ganglioglioma*	I	M	16	nk	0%	0%	0%	0%	0%	0%	0%

Note

The percentage of methylation is expressed with different color intensity (lowest DNA methylation level—brightest color, highest—the darkest one). Indication "nk", not known—indicates samples for which the results were not obtained or were ambiguous.

Tumor samples were collected between January 2010 and September 2013, and they were stored at −80°C. The samples were evaluated at the Laboratory of Neuropathology and grouped according to the histological type and the 2007 WHO classification criteria. Overall survival (OS) time was known for more than half of the patients. All the patients gave informed consent for the analyses to be undertaken, and the study protocol was approved by the Clinical Research Ethics Committee (number 505/12).

### 2.2 DNA isolation and bisulfite conversion

GeneMATRIX Tissue DNA Purification Kit (EurX, Gdańsk, Poland) was used for DNA isolation. DNA concentration and purity were verified using NanoDrop spectrophotometer. Bisulfite conversion of 500 ng of genomic DNA was performed using EZ DNA Methylation Kit (Zymo Research, USA), following the manufacturer's protocol. The elution volume after bisulfite conversion was 10 µL, and 1 µL of each converted DNA was taken for the subsequent MS‐HRM analysis.

### 2.3 Primer sequences and design

Data on primers for *SFRP1*, *SFRP2*, *RUNX3*, *MGMT*, and *RASSF1A* were taken from the literature,^36^, ^37^, ^38^, ^39^, ^40^ whereas those for *INA* and *CBLN4* were designed using Methyl Primer Express v 1.0 software (Applied Biosystems) (Figure S1). The synthesis of primers took place at the Institute of Biochemistry and Biophysics, Polish Academy of Sciences, Warsaw, Poland. Their sequences, together with the annealing temperature, number of CpGs evaluated, and amplicon size, are presented in Table [Link], [Link]. Primer design was performed according to the guidelines of Wojdacz et al^39^ and recommendations provided by Applied Biosystems.^41^ Optimal annealing temperatures (60‐65℃), and optimal amplicon length (100‐200 bp), as well as the recommended number of potential methylation sites in the amplicons (3‐22), were considered during primer design.^42^, ^43^, ^44^ Each primer of the two primer pairs also contained 1 CpG site, to increase the binding to methylated DNA and to overcome the PCR bias.

### 2.4 Standards

CpG Methylated HeLa Genomic DNA (New England Biolabs, USA) and CpGenome Universal Unmethylated DNA Set (Merck, Germany) of equal concentration were mixed in different ratios (0%, 5%, 10%, 25%, 50%, 75%, 100% methylated DNA) to mimic DNA samples with defined levels of DNA methylation. These standards were used for the evaluation of the sensitivity of the assay and the semi‐quantitative estimation of gene promoter methylation in the clinical samples. The assays were optimized in terms of primer annealing temperature to obtain the best possible resolution and the highest sensitivity.

Moreover, the whole‐genome amplified DNA from pooled peripheral blood lymphocytes was prepared with GenomePlex^®^ Whole Genome Amplification Kit (Merck, Germany), according to manufacturer's protocol, and was used for each run.

### 2.5 MS‐HRM analysis

MS‐HRM analysis was performed using Light Cycler^®^ 96 (Roche Diagnostics GmbH, Germany). Bisulfite‐converted DNA was amplified using 5x Hot FIREPol EvaGreen HRM Mix (Solis BioDyne Co., Estonia). Reactions were carried out in a total volume of 20 µL containing 5× HOT FIREPol EvaGreen HRM Mix, 10 pmol/µL of each primer, depending on the gene assayed and 1µl of the template. Samples were run in duplicate in each experiment to verify the reproducibility, and each experiment was repeated twice. The protocol involved 15 minutes of preincubation at 95℃ and 40 cycles of three‐step amplification (15 seconds/95℃, 20 seconds/Ta, 20 seconds/72℃), and obtained amplicons were melted in a temperature gradient to max 95℃ (each Ta is presented in Table [Link], [Link]).

The obtained melting curves were normalized automatically by the calculation of the "line of the best fit" in between two normalization regions before and after the significant fluorescence decrease. The methylation level of each sample was assessed by comparison of the PCR product normalized melting curve/peak with the normalized melting curves/peaks of the controls. Data interpretation was performed according to the guidelines of Smith et al.^42^

### 2.6 Pyrosequencing

Pyrosequencing of *MGMT* gene was performed on Pyrosequencer PSQ™ 96 ID system, and the data were analyzed on PyroQ CpG™ software 1.0.9 (Biotage, Uppsala, Sweden), as described previously [28]. With the use of PyroMark™ MGMT Kit (Biotage, Uppsala, Sweden), we analyzed five CpG sites in exon 1 (positions 17 to 39, Ensemble ID: OTTHUMT00000051009) of the *MGMT* promoter. The temperature profile was the following: 95°C for 15 minutes; 45 cycles of 94°C for 30 seconds, 53°C for 30 seconds, and 72°C for 30 seconds; followed by 72°C for 10 minutes. The amplicon length was 104 bp. A cytosine not followed by a guanine, and which is therefore not methylated, served as an internal control for the completion of bisulfite conversion reaction. The mean methylation across all CpG sites analyzed was calculated for each sample and used for comparison with MS‐HRM.

### 2.7 Statistical analysis

All variables were measured on an ordinal scale, so they did not require any test to assess normality distribution. Nonparametric tests, Mann‐Whitney U test, Kruskal‐Wallis, or Wilcoxon tests were used to analyze data, and the results with *P*‐value < 0.05 were considered significant. The descriptive statistics were performed, and methylation levels of selected genes were examined using STATISTICA 13.1 software, depending on patients’ gender, age, survival time, histological type, and the WHO grade of the tumor. Genes were also analyzed in pairs, for the estimation of the interrelation between their methylation profiles. DNA methylation in cancerous and non‐cancerous samples was additionally determined based on GraphPad Instant 3 and PQStat v 1.6.8 statistical tools. It has to be mentioned that WHO grade I tumors were excluded from the statistical analysis due to the low number of samples (2/46). For comparison between MS‐HRM and pyrosequencing results, Cohen's kappa coefficient was applied to measure the ordinal association between measured quantities, when data were dichotomized. Moreover, Kendall's coefficient of concordance was used when the results of both methods were categorized as percent ranges.

## 3 RESULTS

### 3.1 MS‐HRM analysis of a gene panel of promoter DNA methylation

The normalized melting curves and normalized melting peaks of the analyzed genes obtained from standards and representative samples are presented in Figure S2 (*SFRP1)*, Figure S3 (*SFRP2)*, Figure S4 *(RUNX3)*, Figure S5 (*CBLN4)*, Figure S6 *(INA)*, Figure S7 (*MGMT)*, and Figure S8 (*RASSF1A)*. The methylation level of each sample was assessed by comparison of the PCR product and standards melting profiles with a known ratio of methylated and unmethylated templates. The melting profiles were displayed as both, normalized melting curves and normalized melting peaks, to ease the distinction of each sample melting profile characteristics.

Data were analyzed using the "Tm calling" module of Light Cycler 96 SW 1.1 Software. The algorithm used in data calculation provided default settings for the temperature ranges specifying the normalization areas. Premelting signals were uniformly set to a relative value of 100%, while postmelting signals were set to a relative value of 0%.

**FIGURE 1:**
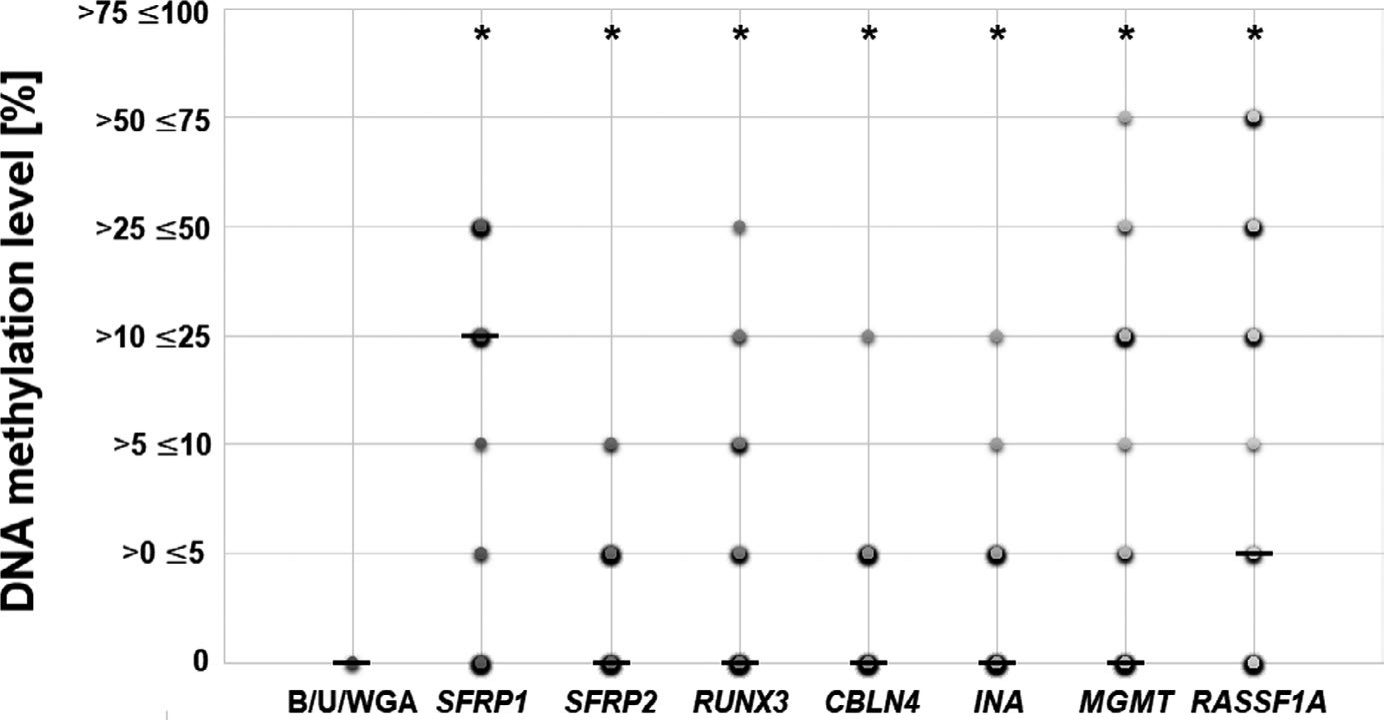
DNA methylation level [%] of the analyzed 7‐gene panel in relation to non‐neoplastic, control brain sample (indicated with "B"), unmethylated DNA control (indicated with "U"), and whole‐genome amplified DNA from pooled peripheral blood lymphocytes (indicated with "WGA"). The size and color intensity of each dot represents the number of samples with the particular methylation range. Median is indicated with a horizontal line "–" whereas * represents statistically significant difference (*P* < 0.05) in regard to the results obtained for sample "B," "U," and "WGA."

The results of the MS‐HRM analysis of all analyzed genes (*SFRP1*, *SFRP2*, *RUNX3*, *CBLN4*, *INA*, *MGMT*, and *RASSF1A*) showed that their promoter sequence methylation level is significantly higher (*P* < 0.0001) in DNA samples from brain tumor patients than in the non‐neoplastic brain sample (Figure 1). The highest possible level of gene promoter methylation (>75, ≤100%) was not reached in any sample or gene, while the methylation level of >50, ≤75% was observed for *RASSF1A* (6/46 samples; 13.04%). The level of >25, ≤50% was achieved frequently for *SFRP1* (12/46 samples; 26.09%) and *RASSF1A* (8/46 samples; 17.39%), followed by *MGMT* (2/46 samples; 4.35%) (Table 1). *RUNX3* was unmethylated in the majority of cases (31/46; 67.39%), but the methylation level of >25, ≤50% was reached in 1 sample, and the level of >10, ≤ 25% was observed in 4 samples, whereas 5 samples were scored as >5, ≤ 10% methylation. More than half of the samples were unmethylated (0% methylation) in regard to *SFRP2* (24/44; 54.54%), while 2 samples reached the level >5, ≤ 10% methylation and 18 samples scored >0, <5% methylation. Both *CBLN4* and *INA* were mostly unmethylated; only single samples reached the level of >10, ≤ 25% methylation.

**FIGURE 2:**
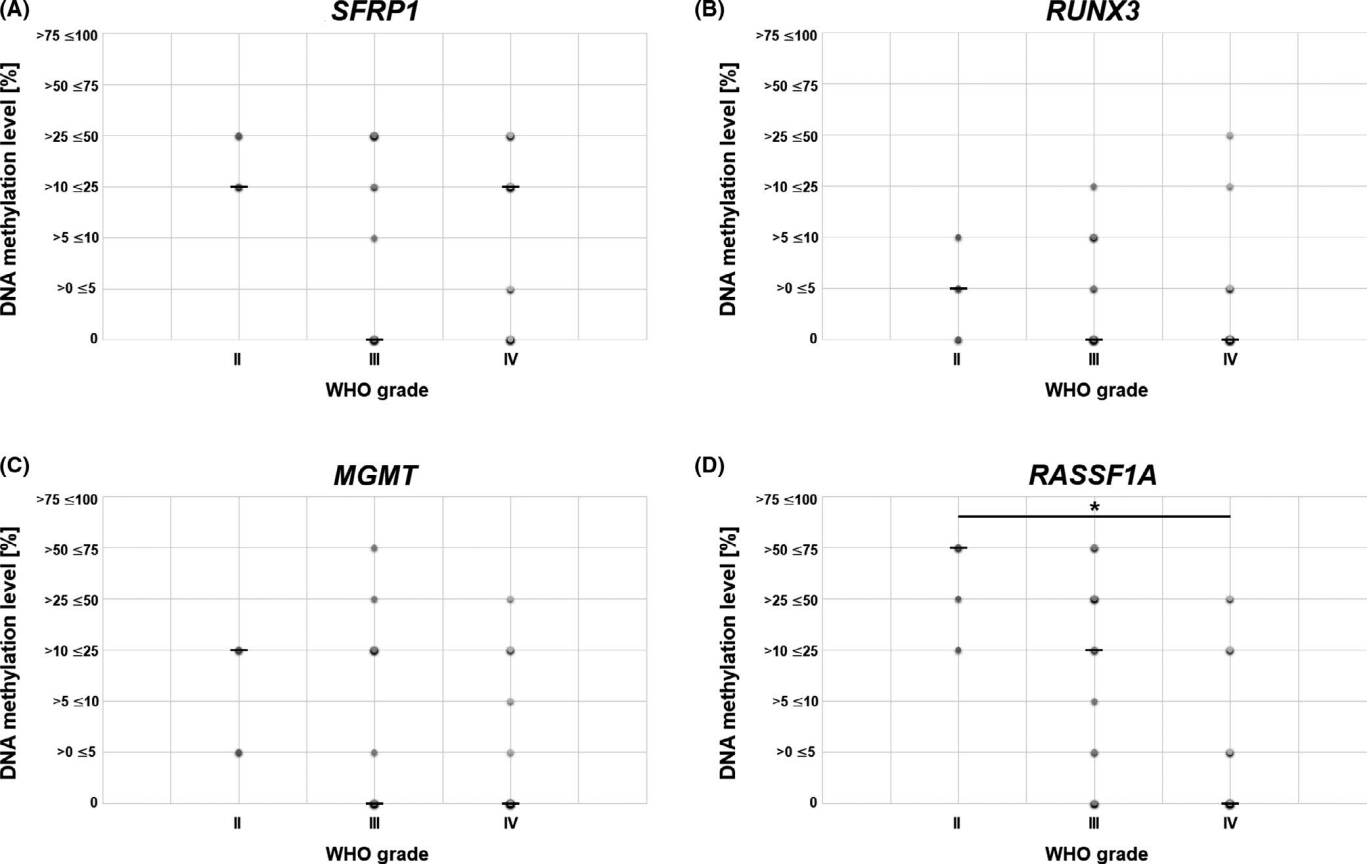
DNA methylation level [%] of *SFRP1*, *RUNX3*, *MGMT*, and *RASF1A* in relation to tumor WHO grade. The size and color intensity of each dot indicates the number of samples representing the particular methylation range. Median is indicated with a horizontal line "–". *RASSF1A* promoter methylation level is significantly higher in lower‐grade gliomas (*P* < 0.05)

**FIGURE 3:**
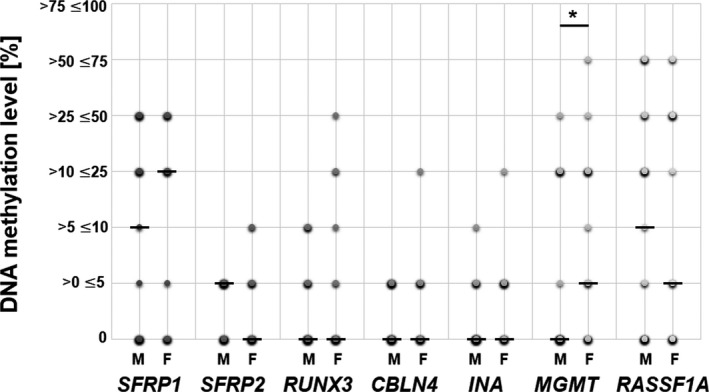
DNA methylation level [%] of the analyzed 7‐gene panel in relation to patients’ gender. M indicates male, F—female. The size and color intensity of each dot indicates the number of samples representing the particular methylation range. Median is indicated with a horizontal line "–". *MGMT* promoter methylation level is significantly higher in female patients, as compared to the value in males (*P* < 0.05)

**FIGURE 4:**
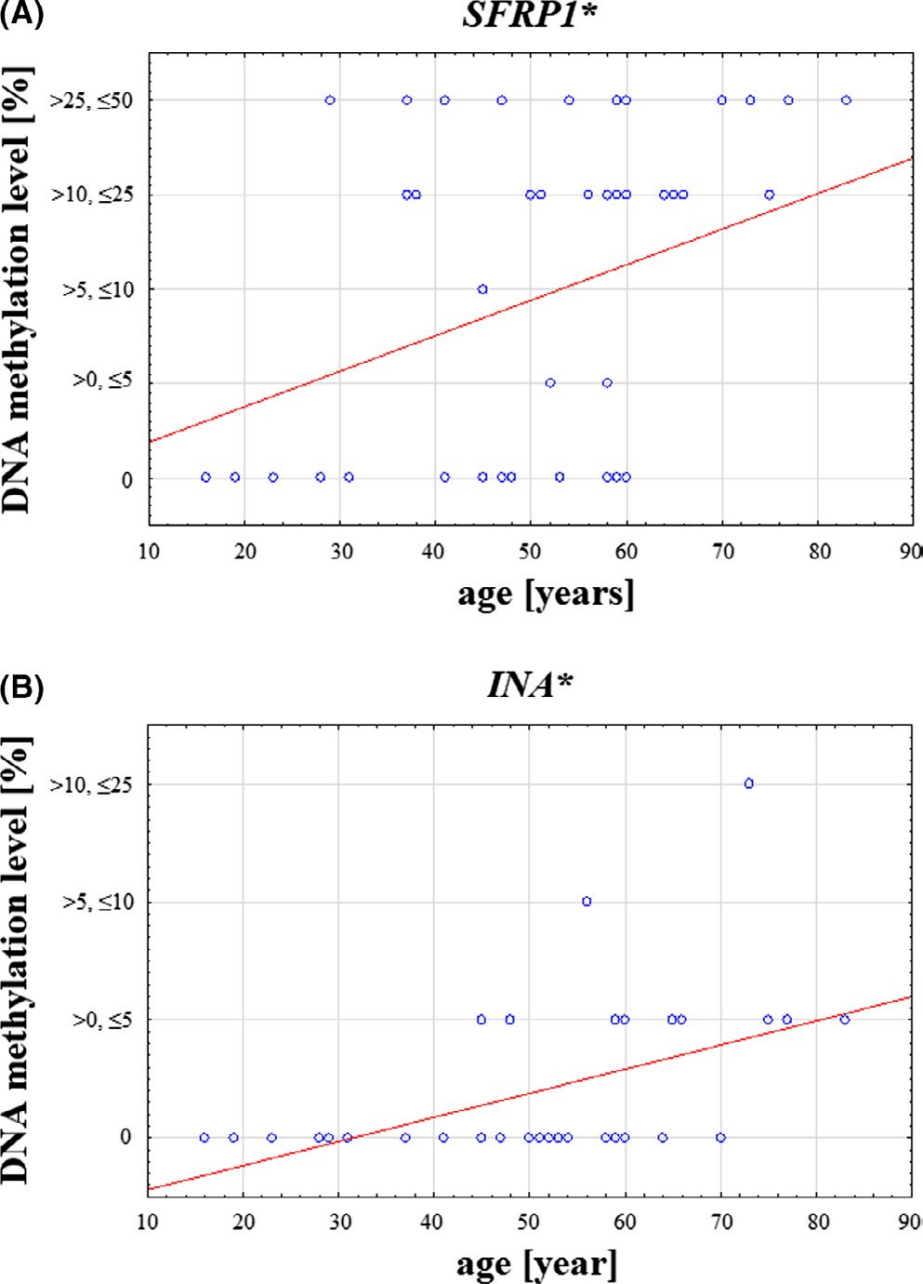
DNA methylation level [%] of *SFRP1* and *INA* in relation to patients’ age [years]. DNA methylation levels of both genes are significantly higher in older patients (*P* < 0.05)

*RASSF1A* had significantly higher methylation level in lower‐grade tumors (Figure 2). A similar relation was observed for *SFRP1*, *MGMT*, and *RUNX3*, but these results did not reach the statistical significance. The distribution of the obtained methylation levels of each gene regarding patients’ gender is presented in Figure 3. The statistical significance (*P* < 0.05) was found between males and females in the case of *MGMT*. The positive correlation of promoter DNA methylation and patients’ age was found in *SFRP1* and *INA* (Figure 4). The analysis of the interrelation between *MGMT*,*SFRP2*, and *RUNX3* methylation levels and patients' age also revealed a trend toward higher methylation levels in older patients, however, without statistical significance (Figure S9). Finally, the overall survival time of patients ranged from 3 to 42 months. The average survival time was 13.5 months, and the median was estimated as 10 months. No correlation was noted between gene promoter methylation level and patients’ survival time (Table S3).

### 3.2 Interrelations between promoter methylation levels of genes in the analyzed panel

**FIGURE 5:**
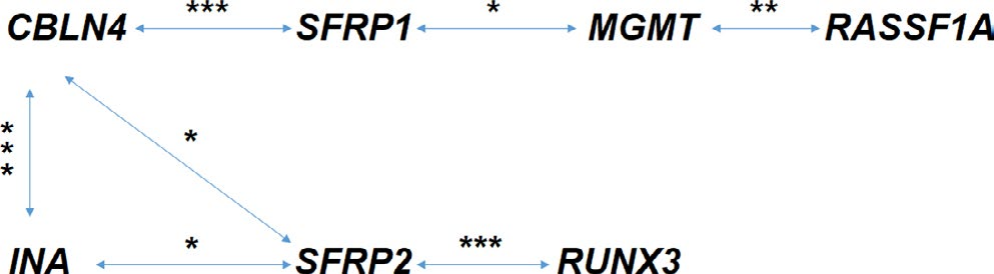
The interrelation between the methylation levels of genes analyzed in pairs. * indicates statistical significance at *P* < 0.05, **P < 0.005, and ***P < 0.0005

The strong interrelationship between promoter methylation levels was detected between pairs of genes: *SFRP1* and *CBLN4* (*P* = 0.00031), *MGMT* and *RASSF1A* (*P* = 0.002337), *SFRP1* and *MGMT* (*P* = 0.035), *SFRP2* and *RUNX3* (*P* = 0.000773), *SFRP2* and *CBLN4* (*P* = 0.049108), *SFRP2* and *INA* (*P* = 0.02783), and *CBLN4* and *INA* (*P* = 0.000137) (Figure 5). For a particular patient, an increased DNA methylation level in one of the paired genes was associated with higher methylation in the other gene promoter, and a lack of methylation in one gene correlated with a lack of methylation in another. This finding proves our concept of choosing the panel of genes which represent different aspects of CNS carcinogenesis, but show relations to each other.

### 3.3 Comparison of MS‐HRM and pyrosequencing of *MGMT* gene

In order to validate the results of MS‐HRM, pyrosequencing of *MGMT* promoter region was applied to all 46 samples analyzed. Ten representative pyrograms and corresponding MS‐HRM normalized melting curves are presented in Figure S10. Both methods were highly reproducible, and when the results of both methods were dichotomized (>10% regarded as methylated, ≤ 10% regarded as unmethylated), they were fully concordant (Cohen's kappa coefficient κ = 1, *P* < 0.000001). There were 15/46 (32.61%) methylated samples and 31/46 (67.39%) unmethylated samples. When pyrosequencing data (mean methylation percentage of five CpG dinucleotides) were grouped into percentage ranges, following MS‐HRM categorization, Kendall's coefficient of concordance was still showing good correlation between the ranges (Kendall's W = 0.92, *P* = 0.00051).

## 4 CONCLUSIONS

New epigenetic biomarkers associated with CNS tumors are constantly being sought and tested. Their identification would enable more rational selections of strategies to cure patients and prevent disease relapse. However, it is already known that probably there cannot be a single gene test for glioma detection, as well as for predictive and prognostic purposes, because of the multifactorial process of carcinogenesis.^17^ Epigenetics is another area for exploring the disease background and biomarkers, as well as the search for potential therapeutic targets.^45^

Additionally, the debate over the best analytical method for methylation markers detection is still ongoing.

We proposed a 7 gene panel for brain glioma characterization. It consists of genes involved in carcinogenesis in general and CNS pathology in particular, as well as having a proven impact on therapeutic response. We confirmed a significantly higher DNA methylation level for each of the 7 analyzed genes in glioma patients compared with the non‐neoplastic brain and unmethylated control. Similar results were obtained by Shinawi et al^31^ and Lai et al,^46^ who differentiated DNA methylation levels in CNS cancer patients and healthy, cancer‐free individuals. Thus, this panel of genes could be used for the differentiation of the CNS tumors from the tissue without signs of tumor growth. Moreover, we observed a significant positive correlation between promoter methylation levels in gene pairs: *SFRP1*/*MGMT*, *SFRP2*/*RUNX3*, *SFRP2*/*CBLN4*, *SFRP2*/*INA*, *CBLN4*/*INA*, and *MGMT*/*RASSF1A*. Increased DNA methylation level in one gene was accompanied by a higher methylation level in another and *vice versa*. Such relationships are valuable markers since they increase screening cost‐effectiveness by narrowing the molecular diagnostic panel of genes.

In this study, the highest median of promoter methylation (>25 ≤50%) was obtained for *RASSF1A* analysis. Interestingly, we observed a general decrease in promoter DNA methylation of *RASSF1A* with increasing glioma malignancy (this effect was also observed to a lesser extent for *SFRP1*, *MGMT*, and *RUNX3*). This result is concordant with previous findings on total DNA demethylation with increasing glioma grade,^47^ being the effect of oxidative DNA damage.^48^ In that context, the interesting aspect is a stable level of *CBLN4*, *INA*, and *SFRP2* promoter methylation, which can make them promising biomarkers of neuro‐oncogenesis in general.

Reports published to date provide moderate evidence of the correlation between DNA methylation level and the CNS cancer histological type. Gao et al^49^ examined 28 samples of glial tumors. They found that *RASSF1A* hypermethylation is associated with a loss of expression, but it is not statistically significant in regard to histological type and malignancy of CNS tumors. In turn, Muñoz et al^50^ observed the relationship between *RASSF1A* promoter methylation and a secondary glioma phenotype. In our previous study, we showed that *RASSF1A* hypermethylation differentiated primary from metastatic brain cancers.^35^ Thus, this gene should further be tested and validated in regard to CNS tumors.

Among many assays for DNA methylation analysis, MS‐HRM is one of the most frequently recommended methods.^13^, ^14^ It has already been proved useful in the assessment of cancer biomarkers in bladder, colorectal, and breast cancer patients.^11^ Our study is the first report presenting the application of MS‐HRM for CNS tumor analysis. One of the possible disadvantages of MS‐HRM is that the reaction is semi‐quantitative since the results of the analysis are presented as a methylation range. Nevertheless, such data presentation is commonly regarded as sufficient for the sample evaluation in the clinics.^51^ Many advantages of the method, including its sensitivity as well as specificity, cost‐effectiveness, and no sophisticated equipment needed, make this method suitable for the routine clinical application. The fact that MS‐HRM analysis requires no manual post‐PCR processing and is performed in a closed‐tube system, with minimal risk of contamination, is equally important.

In order to validate MS‐HRM results, we performed additional pyrosequencing analysis of the most important biomarker from our panel, namely *MGMT*. For the purpose of this comparison, the pyrosequencing data were categorized into methylation ranges, same as in MS‐HRM method. The obtained data showed high agreement among methylation ranges, even though both methods do not analyze exactly the same CpG dinucleotides (Figure S10). However, when pyrosequencing and MS‐HRM results were dichotomized into methylated and unmethylated groups (taking 10% methylation as a cutoff value) the results were fully concordant. Similar results were reported by several groups.^52^, ^53^, ^54^

In this study, we also established and optimized protocols for MS‐HRM analysis of *CBLN4* and *INA* genes, which were, according to our best knowledge, not reported so far. The reaction was optimized in terms of, for example, primer sequences and primer annealing temperature, so that the fluorescent melting profiles from subsequent methylated DNA standards exhibit significant differences. The sensitivity of both assays was high, allowing the detection of as low as 5% methylation. Indeed, in our tested group, the fluorescent curves of almost all of the samples were found between 0% and 5% methylated standards. *INA* encodes a protein that is a member of the intermediate filament family and is involved in the morphogenesis of neurons. It is a novel candidate for a CNS biomarker, indicating GBM with a worse prognosis.^31^ In turn, *CBLN4* encodes a cerebellin 4 precursor, involved in the regulation of neurexin signaling during synapse development.^55^ Their clinically relevant level of promoter methylation and potential role as CNS cancer biomarkers should be determined in further studies.

In the current study, not only *INA* and *CBLN4* but also other genes from the chosen panel did not show any association between DNA methylation level and patients’ OS. Thus, their prognostic potential is minimal. However, in our previous study, *SFRP1* methylation was associated with shorter OS.^26^ Also, *MGMT* methylation status represents one of the most relevant prognostic factors in GBMs.^4^, ^33^ Therefore, the results of our study should be verified on larger patients' cohorts.

CNS tumors have two morbidity peaks. The first one occurs in childhood, and the second one is between 55 and 65 years of age.^56^ In this study, patients’ age ranged between 16 and 83 years of age, and higher methylation levels of two genes, namely *SFRP1* and *INA*, were observed in older patients, which confirmed our previous observation,^26^ indicating a more frequent occurrence of *SFRP1* ^26^ and *RUNX3* ^27^, ^28^ promoter methylation in older CNS tumor patients. In turn, lack of association between age and *SFRP1* methylation level was proved by Chang^57^ and Kafka,^58^ while no correlation for *MGMT* in the analysis of 58 anaplastic astrocytomas was reported by Bell et al.^59^

The patient's sex is another recognized prognostic factor for brain malignancies. Epidemiological data show that glial tumors are slightly more common (about 1.2×) in men than women.^56^ In our study, *RASSF1A* was the most frequently methylated gene in both female and male patients. This observation was also confirmed by Muñoz et al^50^ and in our previous studies.^27^, ^28^ Moreover, the results of our current study, showing higher *MGMT* promoter methylation in female as compared to male patients, are in line with Franceschi et al^60^ results. Moreover, in this study, also the patients' sex and methylated *MGMT* occurrence were significantly related to patients' survival time. CNS cancer female patients with methylated DNA lived significantly longer as compared to men with the methylated *MGMT*, showing the importance of sex as a prognostic factor.^60^ Tian et al^61^ also confirmed the prognostic significance of sex in women with GBM. Therefore, the role of female hormones in glial tumor pathogenesis should be further clarified.

The development of diagnostic methods and discovering new biomarkers gain importance in neuro‐oncology.^62^, ^63^ It is necessary to introduce epigenetic biomarker panels for CNS tumors, enabling earlier cancer detection or better therapy monitoring. Our study verifies MS‐HRM usefulness for the assessment of DNA methylation in CNS tumors. We conclude that our 7‐gene panel of promoter methylation can be helpful in brain tumor diagnosis or characterization, and MS‐HRM can play a crucial role in the development of cost‐efficient personalized patient care.

## CONFLICT OF INTEREST

The authors declare no conflict of interest.

## Supporting information

Fig S1

Fig S2

Fig S3

Fig S4

Fig S5

Fig S6

Fig S7

Fig S8

Fig S9

Fig S10

Table S1

Table S2

Table S3
