# Supplementary material for: DNA methylation analysis with methylation‐sensitive high‐resolution melting (MS‐HRM) reveals gene panel for glioma characteristics
Source: CNS Neurosci Ther. 2020 Aug 11;26(12):1303–14. doi: 10.1111/cns.13443 (PMC7702229; doi:10.1111/cns.13443)
Supplement: Supplementary file 1 — Fig S1 [file CNS-26-1303-s001.docx]

***CBLN4***

INITIAL NUCLEOTIDE SEQUENCE

ACGACGTCCAGGGACCAGGTGCCCTTCGGCTCCCGCCGACCTGTTGCTCGAAACTTGCCCTGAGCTCTCGCTGCCGGGCTCTGGGCTCCCAAGCCTCTCCCCCGCCGCCGCAGCAGCCTCTTTTAGGGGCCCGGAAGAAATGGGAGCCGGGGCTGGTGAGAGGGGTAGGAAGAGGGACGGTAGAAGTTTCAGACCCAGGCATATTTGGGAAGGCGAGTGCTTTACATGATTCCCCATTTCCCAATCGGACCAGCTTAGCCTGGGCAGGCAGCCTCGTGCTGAGGTGCCTGGAGACCGCCCACCCCCTAGGTGCTCGCTTCCCCCCGGGTCTGACCTGATCGAAGTAAATGATGCGCGTCTTGTTGCTCATCTCGGATGGCTCGTGGTTGGTGCTCCGCACCGCCGAGAAGGCGACCTTGGAGTTGGCCGCCCGGACCGATATCCCCAGCGGGGAGGAAGAGGAGCCCTTGGAGTCCGTGGCCGGGTTCGAGTCGCACACCACCAGACACTTGCCCTCCAGCACGATGGGCTCCGTGTCGTTCTGTGCCCAGACGGGCAGCCCCGGCAGCGTGAGGACCAGCAGCACGGCCGGCACCGCGGACAGCGCCCGGCGCCCGGAGCCCATGGTGAGCCGTGTGGGCAGCCGCAGCCGGCTGGCGCTGGTGCTCGCCCGCGTCGCCTCCTACCCCGGGATCCCGGTGCTCGGGAAGATGCTAGCGGCTAGGTCGACAGCGCTGCAGGAGCGACGGCGGCGGCGGCGCGCACACTTCCACCAATTCTGTGGCTTGAAGTCAAAGTCTCCCCTCGAGCTCTCTCGCTGGCTCTGTTACCTTTGTCCTTTAAGGAGCTCATGCAGCACCCTTTACCCTACTCTCCTCCGCCCAAGAATCAGCCCTGCCTGGGGCCCCTGCACCCACTCTGGTTCCTAGACATCTGAAAGTCATCAAACCCTCACATTCACACCTCAAGGCAAAAAATAATAATAATAATAAATTCTCACCCCAAACTCAAGCACCACCAGCTAAACCACGGAGCAGGAACAAAAAGAGGGGACTCAAAGAGAAGCCACAAGGGTGGCGGGTGCCCAGCGGCGCGGGTGCCAGTCCTGTCTGGCTTGCGGCAGGGACGAGTTACAGAGGCAGAAGGTCCTTCCCAGGCTGAGAAGAACGCGAGGCTGTGTTCATGGCCAGGACGCCAGCGACTCCCACTTTCGCCTGGTCAAAAAAATCCCCCAAACCTGGTGTCACCCAGAGGTAGGGAGGGAGGCAGCGGCTAGCCAGGTCCCTCGCACCGAAAGCGCGGATTCGCAGGATCAGGTCCAGGGCGCCGGGCGCAGCGTTCAGGGCGGCTGGTCCGGCGGGGTCCTCTCCTGCCTGGCCGCCCGCCCCCAGTCCCTGCGCACAACTTTCTCGTCCCTCGTGCAGCCCGGAGAGCGCGAAGCGGGCACACGCGCTCTATTTATAGGAGCGCAGCGTCCGGCTGGGTTGGCTTATCGCGCAACCCGCGGGCTCCGAGAAAAGGGGGAGAAGGCGTCTGGTGACCCCATCTGAGCAGCTCTCTCCTGACGTTTAACGCACCCCAAAGCAAGCGGGGCGCACAGCACTGGTGATTGGCAAGGTTCGAGGCCCCTCAGCCCCTGAAAGCCCGTGGCGTAAAGGATGCGTGCCCAGAGAGGAGCCCACGGCCCTGCGCTGGCCCCAGCTCCGCGCCCACTCTCTCCTGCTCGATCTGAGTCCGCAGAGGAGGCGGCTGGTCCCCTCACCCCTCCCAGCGCGGAACTCTGCCGCCGAGTGCCCTCCAAGCGGAGCGCGGCGCTGCCAGGCCGGGTCGCGAAAAAGGATAAGCCGCCGCGGACCCGCGCTGCGCGCTGCCCCGAACCTGGGGACCGGCGGGCGTCCCGACCGCGCCTGGCCGGGAGCCCGCCCCACACAGCCCTGGGGCCTG**GCGAGCTCAGGGAAGCCTTG**GGGCGGACCAGAGCCCGTGGGGGCGGCTGGGAGGAGGTGCCTGATTCCTTCCTTCCGCTCCG**GGAAGATGAGCCTCAGAAGCCGCAG**GGGCGCGCCTTCCCCCCA

BISULFITE MODIFICATION OF DNA

ACGACGTTTAGGGATTAGGTGTTTTTCGGTTTTCGTCGATTTGTTGTTCGAAATTTGTTTTGAGTTTTCGTTGTCGGGTTTTGGGTTTTTAAGTTTTTTTTTCGTCGTCGTAGTAGTTTTTTTTAGGGGTTCGGAAGAAATGGGAGTCGGGGTTGGTGAGAGGGGTAGGAAGAGGGACGGTAGAAGTTTTAGATTTAGGTATATTTGGGAAGGCGAGTGTTTTATATGATTTTTTATTTTTTAATCGGATTAGTTTAGTTTGGGTAGGTAGTTTCGTGTTGAGGTGTTTGGAGATCGTTTATTTTTTAGGTGTTCGTTTTTTTTCGGGTTTGATTTGATCGAAGTAAATGATGCGCGTTTTGTTGTTTATTTCGGATGGTTCGTGGTTGGTGTTTCGTATCGTCGAGAAGGCGATTTTGGAGTTGGTCGTTCGGATCGATATTTTTAGCGGGGAGGAAGAGGAGTTTTTGGAGTTCGTGGTCGGGTTCGAGTCGTATATTATTAGATATTTGTTTTTTAGTACGATGGGTTTCGTGTCGTTTTGTGTTTAGACGGGTAGTTTCGGTAGCGTGAGGATTAGTAGTACGGTCGGTATCGCGGATAGCGTTCGGCGTTCGGAGTTTATGGTGAGTCGTGTGGGTAGTCGTAGTCGGTTGGCGTTGGTGTTCGTTCGCGTCGTTTTTTATTTCGGGATTTCGGTGTTCGGGAAGATGTTAGCGGTTAGGTCGATAGCGTTGTAGGAGCGACGGCGGCGGCGGCGCGTATATTTTTATTAATTTTGTGGTTTGAAGTTAAAGTTTTTTTTCGAGTTTTTTCGTTGGTTTTGTTATTTTTGTTTTTTAAGGAGTTTATGTAGTATTTTTTATTTTATTTTTTTTCGTTTAAGAATTAGTTTTGTTTGGGGTTTTTGTATTTATTTTGGTTTTTAGATATTTGAAAGTTATTAAATTTTTATATTTATATTTTAAGGTAAAAAATAATAATAATAATAAATTTTTATTTTAAATTTAAGTATTATTAGTTAAATTACGGAGTAGGAATAAAAAGAGGGGATTTAAAGAGAAGTTATAAGGGTGGCGGGTGTTTAGCGGCGCGGGTGTTAGTTTTGTTTGGTTTGCGGTAGGGACGAGTTATAGAGGTAGAAGGTTTTTTTTAGGTTGAGAAGAACGCGAGGTTGTGTTTATGGTTAGGACGTTAGCGATTTTTATTTTCGTTTGGTTAAAAAAATTTTTTAAATTTGGTGTTATTTAGAGGTAGGGAGGGAGGTAGCGGTTAGTTAGGTTTTTCGTATCGAAAGCGCGGATTCGTAGGATTAGGTTTAGGGCGTCGGGCGTAGCGTTTAGGGCGGTTGGTTCGGCGGGGTTTTTTTTTGTTTGGTCGTTCGTTTTTAGTTTTTGCGTATAATTTTTTCGTTTTTCGTGTAGTTCGGAGAGCGCGAAGCGGGTATACGCGTTTTATTTATAGGAGCGTAGCGTTCGGTTGGGTTGGTTTATCGCGTAATTCGCGGGTTTCGAGAAAAGGGGGAGAAGGCGTTTGGTGATTTTATTTGAGTAGTTTTTTTTTGACGTTTAACGTATTTTAAAGTAAGCGGGGCGTATAGTATTGGTGATTGGTAAGGTTCGAGGTTTTTTAGTTTTTGAAAGTTCGTGGCGTAAAGGATGCGTGTTTAGAGAGGAGTTTACGGTTTTGCGTTGGTTTTAGTTTCGCGTTTATTTTTTTTTGTTCGATTTGAGTTCGTAGAGGAGGCGGTTGGTTTTTTTATTTTTTTTAGCGCGGAATTTTGTCGTCGAGTGTTTTTTAAGCGGAGCGCGGCGTTGTTAGGTCGGGTCGCGAAAAAGGATAAGTCGTCGCGGATTCGCGTTGCGCGTTGTTTCGAATTTGGGGATCGGCGGGCGTTTCGATCGCGTTTGGTCGGGAGTTCGTTTTATATAGTTTTGGGGTTTG**GCGAGTTTAGGGAAGTTTTG**GGGCGGATTAGAGTTCGTGGGGGCGGTTGGGAGGAGGTGTTTGATTTTTTTTTTTCGTTTCG**GGAAGATGAGTTTTAGAAGTCGTAG**GGGCGCGTTTTTTTTTTA

FORWARD

**Length:** 20bp.

5' GYGAGTTTAGGGAAGTTTTG 3'

Tm=60.22; CpG=1; C=4

**You may modify the primer sequence if necessary, within this region:**

5' TAGTTTTGGGGTTTGGYGAGTTTAGGGAAGTTTTGGGGYGGATTAGAGTT 3'

REVERSE

Length: 25 bp.

5' CTACRACTTCTAAAACTCATCTTCC 3'

Tm=59.89; CpG=1; C=5

**You may modify the primer sequence if necessary, within this region:**

5' AAAAAAAACRCRCCCCTACRACTTCTAAAACTCATCTTCCCRAAACRAAAAAAAA 3'

PCR PRODUCT

**Length:** 107 bp.

5' GYGAGTTTAGGGAAGTTTTGGGGYGGATTAGAGTTYGTGGGGGYGGTTGGGAGGAGGTGTTTGATTTTTTTTTTTYGTTTYGGGAAGATGAGTTTTAGAAGTYGTAG 3'

%CGs=46.73

Chromosome 20: 56,021,019-56,021,127

***INA***

INITIAL NUCLEOTIDE SEQUENCE

TCACTTCCGAAGCAGTCGATTCTTGGGGAGAAGCGCTGCGGAAAGGGGCGACTCCGATGCAGATGGCCCTGTCCCGGCGCCCCAGGTCGTCGCGCGCGCAGCTGCGGTAGTCACTGCGCCTCCCCGCCCCCACTCCTGGATGCCCCCCTTCCCTCTCCCGGCCAGACTCTGAGCAGGAGCTCCGCCCCCAGCGCGCCGCCCCAGCCCCGGCGCCTTAAAAGCCGGGCGCACCGCCCCGCCGCGCCCTGCCTGCCGCACCTCTCCTTTCTTCTGTAGCTCGCGTTGAAGCCGCACGTCCGGCCCCGATCCCGGCACCATGAGCTTCGGCTCGGAGCACTACCTGTGCTCCTCCTCCTCCTACCGCAAGGTGTTCGGGGATGGCTCTCGCCTGTCCGCCCGCCTCTCTGGGGCCGGCGGCGCGGGCGGCTTCCGCTCGCAGTCGCTGTCCCGCAGCAATGTGGCCTCCTCGGCCGCCTGCTCCTCGGCCTCGTCGCTCGGCCTCGGCCTGGCCTATCGCCGGCCGCCGGCGTCCGACGGGCTGGACCTGAGCCAGGCGGCGGCGCGCACCAACGAGTACAAGATCATCCGCACCAACGAGAAGGAGCAGCTGCAGGGCCTCAACGACCGCTTCGCCGTGTTCATCGAGAAGGTGCATCAGCTGGAGACGCAGAACCGCGCGTTGGAGGCCGAGCTGGCCGCGCTGCGACAGCGCCACGCTGAGCCGTCGCGCGTCGGCGAGCTCTTCCAGCGCGAGCTGCGCGACCTGCGCGCGCAGCTGGAGGAGGCCAGCTCGGCTCGCTCGCAGGCCCTGCTGGAGCGCGACGGGCTGGCGGAGGAGGTGCAGCGGCTGCGGGCGCGCTGCGAGGAGGAGAGCCGCGGACGCGAAGGCGCCGAGCGCGCCCTGAAGGCGCAGCAGCGCGACGTGGACGGCGCCACGCTG**GCCCGCCTGGACCTGGAGA**AGAAGGTGGAGTCGCTGCTGGACGAGCTGGCCTTCGTACGCCAGGTGCACGAC**GAGGAGGTAGCCGAGCTGCTG**GCCACGCTGCAGGCGTCGTCGCAGGCCGCGGCCGAGGTGGACGTGACTGTGGCTAAACCAGACCTGACCTCGGCTCTGAGGGAGATCCGCGCCCAGTATGAGTCCCTGGCCGCTAAGAACCTGCAGTCCGCGGAAGAATGGTACAAGTCCAAGTTTGCCAACCTGAACGAGCAGGCGGCGCGCAGCACCGAGGCCATCCGGGCCAGCCGCGAGGAGATCCACGAGTATCGGCGCCAGCTGCAGGCGCGCACCATCGAGATCGAGGGCCTGCGCGGGGCCAACGAGTCCTTGGAGAGGCAGATCCTGGAGCTGGAGGAGCGGCACAGTGCCGAGGTAGCTGGCTACCAGGTAAGGGCCGGGGCTGGGCGTGGGGAGGGGTGCCCTGCCCTCTTCCGCGCGTACCCT

BISULFITE MODIFICATION OF DNA

TTATTTTCGAAGTAGTCGATTTTTGGGGAGAAGCGTTGCGGAAAGGGGCGATTTCGATGTAGATGGTTTTGTTTCGGCGTTTTAGGTCGTCGCGCGCGTAGTTGCGGTAGTTATTGCGTTTTTTCGTTTTTATTTTTGGATGTTTTTTTTTTTTTTTTCGGTTAGATTTTGAGTAGGAGTTTCGTTTTTAGCGCGTCGTTTTAGTTTCGGCGTTTTAAAAGTCGGGCGTATCGTTTCGTCGCGTTTTGTTTGTCGTATTTTTTTTTTTTTTTGTAGTTCGCGTTGAAGTCGTACGTTCGGTTTCGATTTCGGTATTATGAGTTTCGGTTCGGAGTATTATTTGTGTTTTTTTTTTTTTTATCGTAAGGTGTTCGGGGATGGTTTTCGTTTGTTCGTTCGTTTTTTTGGGGTCGGCGGCGCGGGCGGTTTTCGTTCGTAGTCGTTGTTTCGTAGTAATGTGGTTTTTTCGGTCGTTTGTTTTTCGGTTTCGTCGTTCGGTTTCGGTTTGGTTTATCGTCGGTCGTCGGCGTTCGACGGGTTGGATTTGAGTTAGGCGGCGGCGCGTATTAACGAGTATAAGATTATTCGTATTAACGAGAAGGAGTAGTTGTAGGGTTTTAACGATCGTTTCGTCGTGTTTATCGAGAAGGTGTATTAGTTGGAGACGTAGAATCGCGCGTTGGAGGTCGAGTTGGTCGCGTTGCGATAGCGTTACGTTGAGTCGTCGCGCGTCGGCGAGTTTTTTTAGCGCGAGTTGCGCGATTTGCGCGCGTAGTTGGAGGAGGTTAGTTCGGTTCGTTCGTAGGTTTTGTTGGAGCGCGACGGGTTGGCGGAGGAGGTGTAGCGGTTGCGGGCGCGTTGCGAGGAGGAGAGTCGCGGACGCGAAGGCGTCGAGCGCGTTTTGAAGGCGTAGTAGCGCGACGTGGACGGCGTTACGTTG**GTTCGTTTGGATTTGGAGA**AGAAGGTGGAGTCGTTGTTGGACGAGTTGGTTTTCGTACGTTAGGTGTACGAC**GAGGAGGTAGTCGAGTTGTTG**GTTACGTTGTAGGCGTCGTCGTAGGTCGCGGTCGAGGTGGACGTGATTGTGGTTAAATTAGATTTGATTTCGGTTTTGAGGGAGATTCGCGTTTAGTATGAGTTTTTGGTCGTTAAGAATTTGTAGTTCGCGGAAGAATGGTATAAGTTTAAGTTTGTTAATTTGAACGAGTAGGCGGCGCGTAGTATCGAGGTTATTCGGGTTAGTCGCGAGGAGATTTACGAGTATCGGCGTTAGTTGTAGGCGCGTATTATCGAGATCGAGGGTTTGCGCGGGGTTAACGAGTTTTTGGAGAGGTAGATTTTGGAGTTGGAGGAGCGGTATAGTGTCGAGGTAGTTGGTTATTAGGTAAGGGTCGGGGTTGGGCGTGGGGAGGGGTGTTTTGTTTTTTTTCGCGCGTATTTT

FORWARD

**Length:** 19bp.

5' GTTYGTTTGGATTTGGAGA 3'

Tm=60.27; CpG=1; C=6

**You may modify the primer sequence if necessary, within this region:**

5' GAYGGYGTTAYGTTGGTTYGTTTGGATTTGGAGAAGAAGGTGGAGTYGT 3'

REVERSE

Length: 21 bp.

5' CAACAACTCRACTACCTCCTC 3'

Tm=60.43; CpG=1; C=3

**You may modify the primer sequence if necessary, within this region:**

5' CRCCTACAACRTAACCAACAACTCRACTACCTCCTCRTCRTACACCTAACR 3'

PCR PRODUCT

**Length:** 93 bp.

5' GTTYGTTTGGATTTGGAGAAGAAGGTGGAGTYGTTGTTGGAYGAGTTGGTTTTYGTAYGTTAGGTGTAYGAYGAGGAGGTAGTYGAGTTGTTG 3'

%CGs=48.39
