# Supplementary figures and images for: DNA methylation analysis with methylation‐sensitive high‐resolution melting (MS‐HRM) reveals gene panel for glioma characteristics
Source: CNS Neurosci Ther. 2020 Aug 11;26(12):1303–14. doi: 10.1111/cns.13443 (PMC7702229; doi:10.1111/cns.13443)

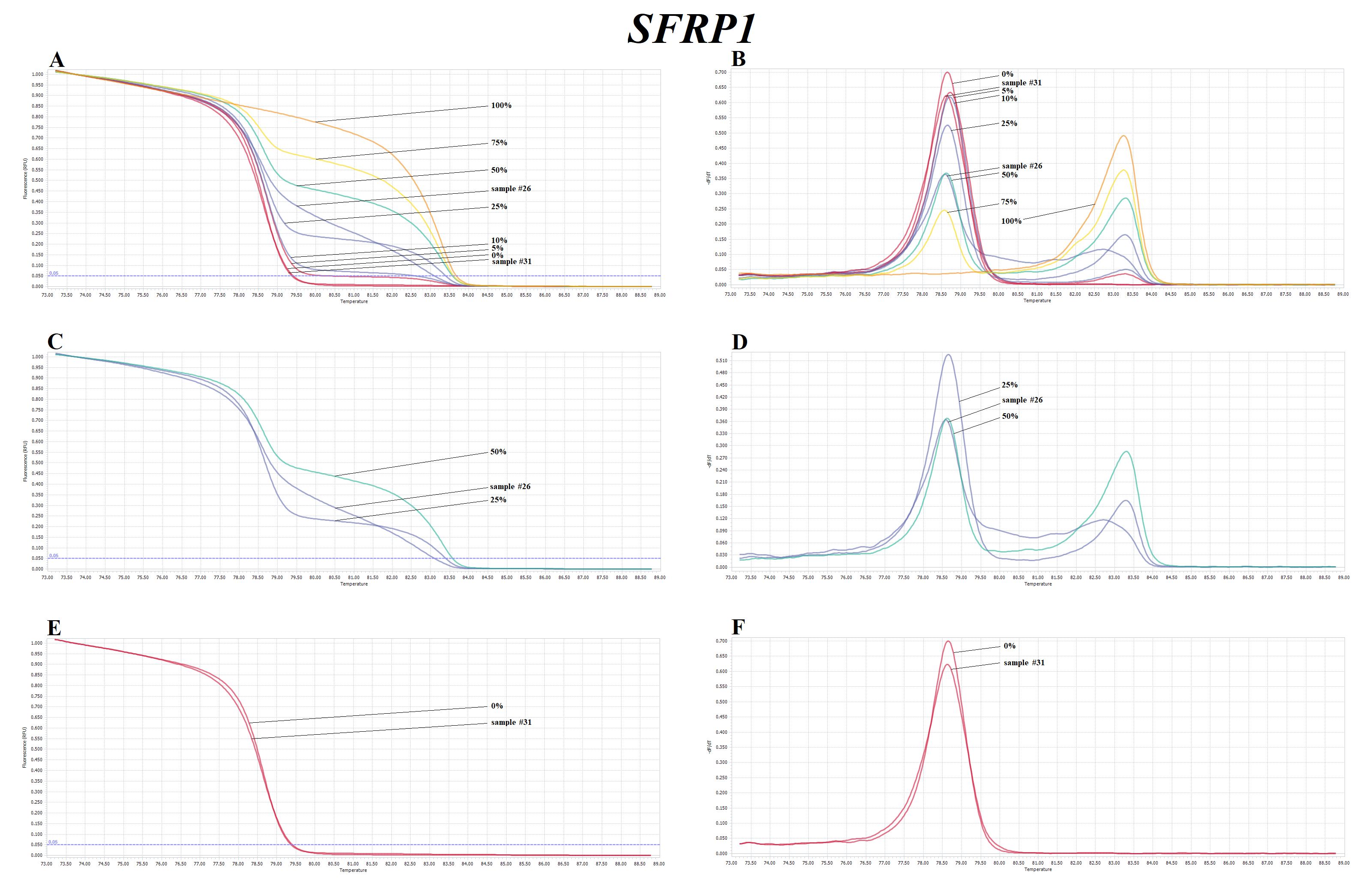

Supplement: Supplementary file 2 — Fig S2 [file CNS-26-1303-s002.tif]

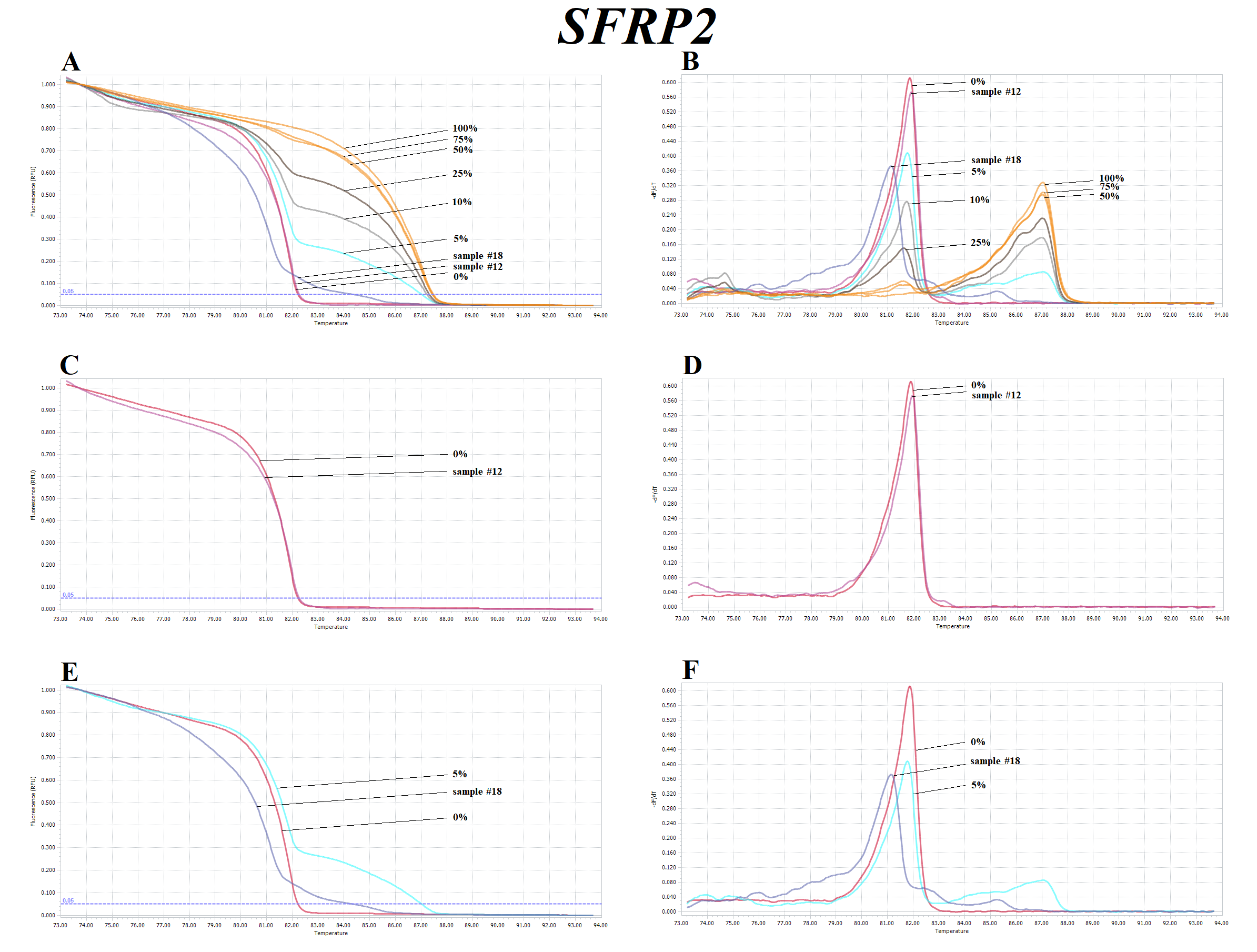

Supplement: Supplementary file 3 — Fig S3 [file CNS-26-1303-s003.tif]

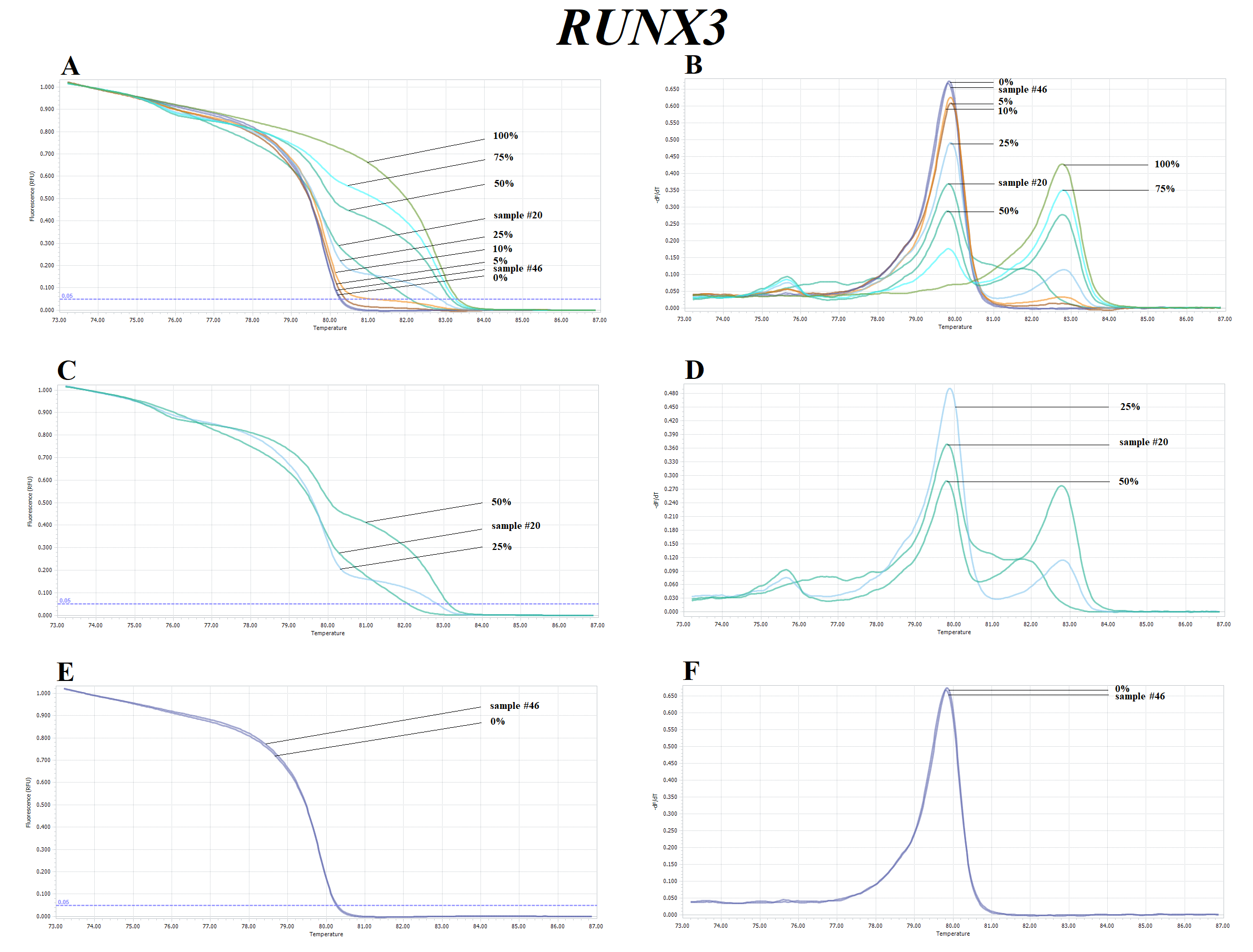

Supplement: Supplementary file 4 — Fig S4 [file CNS-26-1303-s004.tif]

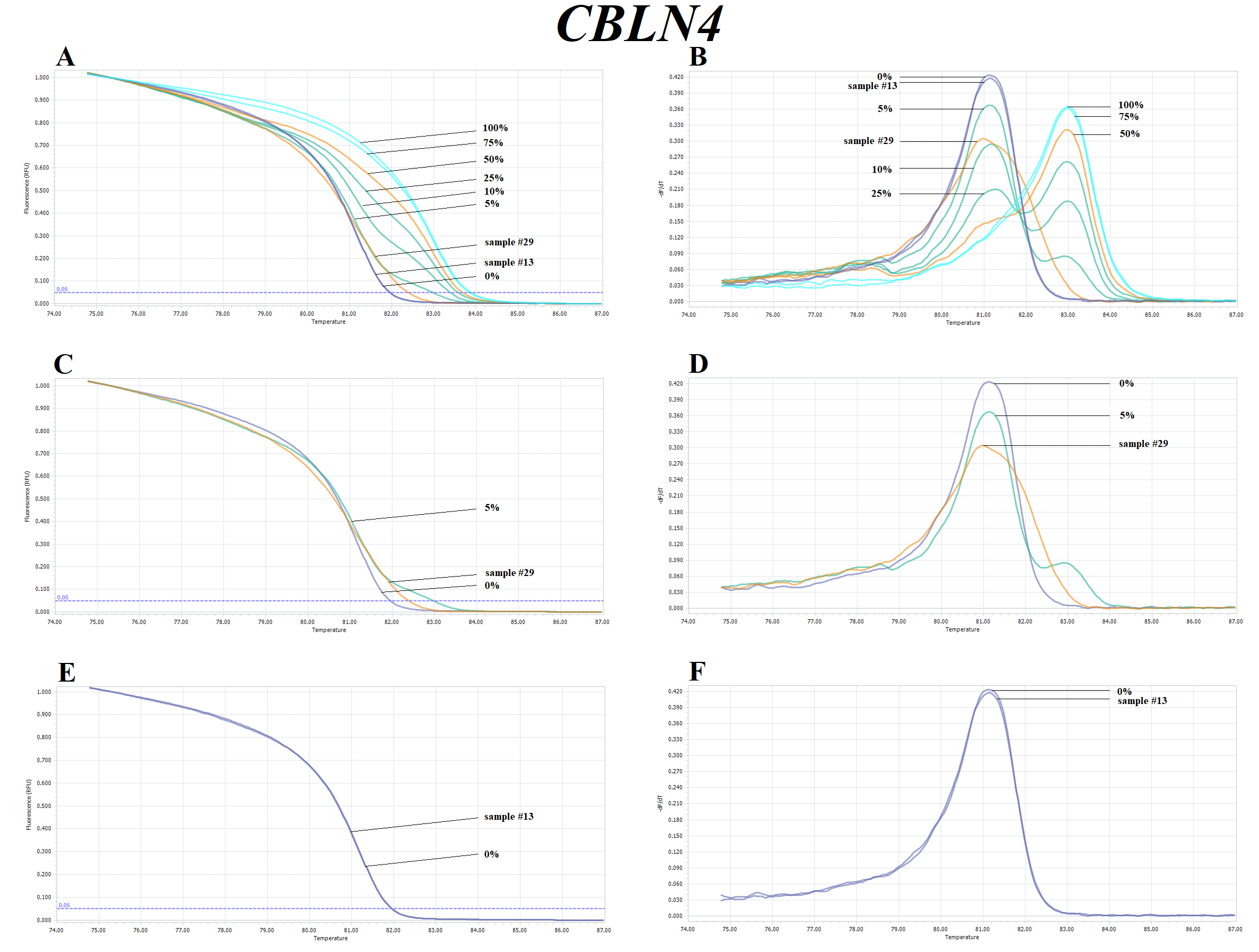

Supplement: Supplementary file 5 — Fig S5 [file CNS-26-1303-s005.tif]

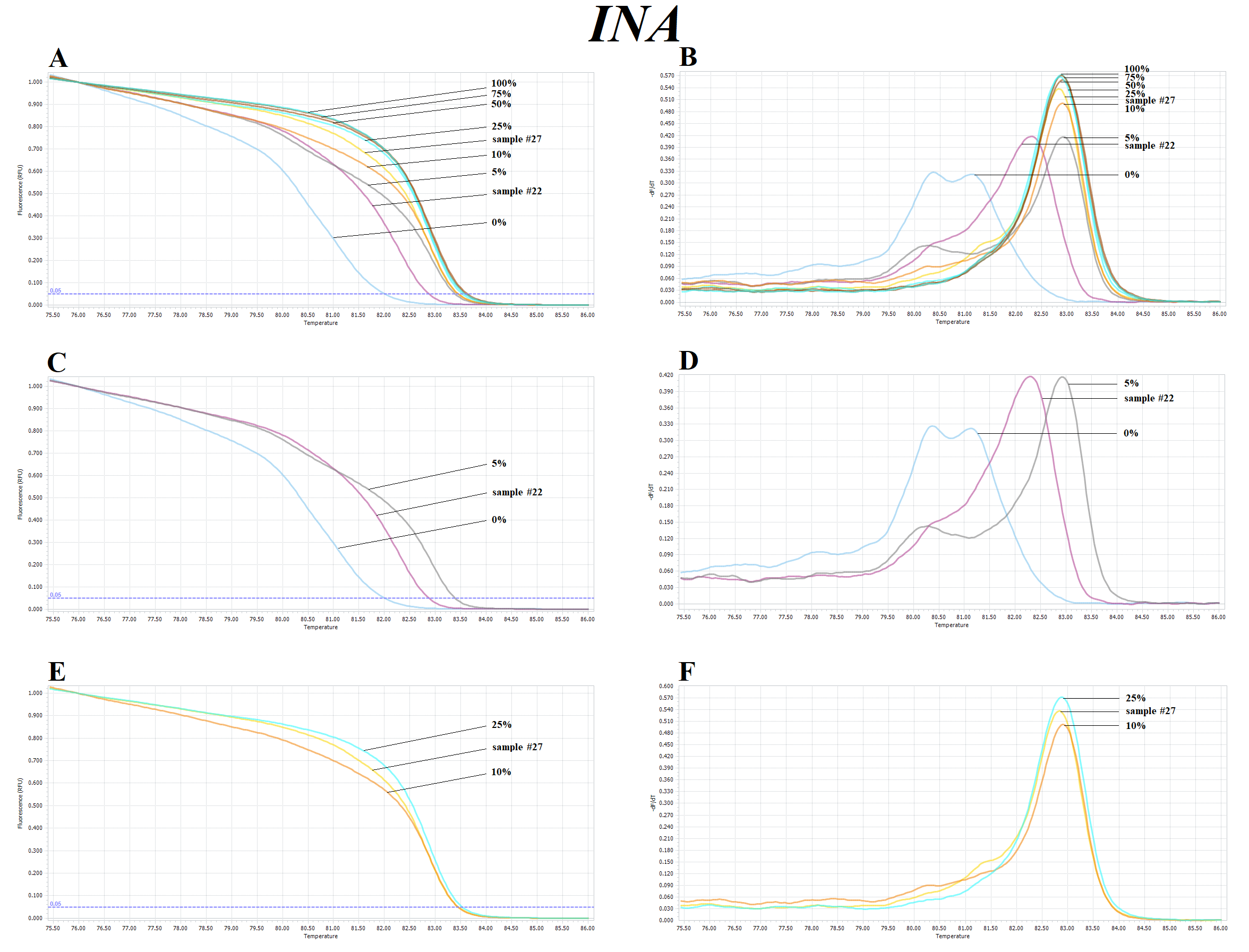

Supplement: Supplementary file 6 — Fig S6 [file CNS-26-1303-s006.tif]

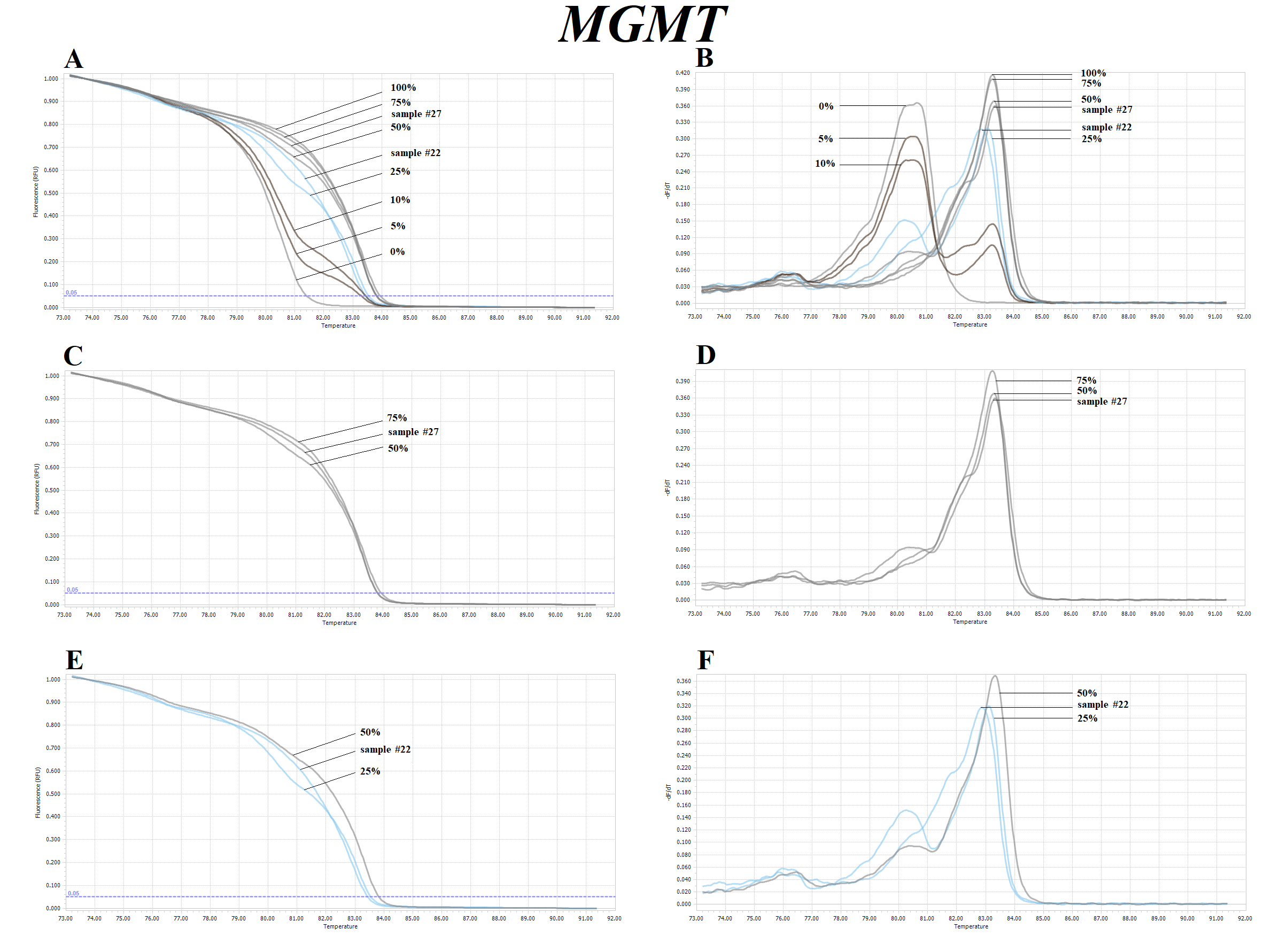

Supplement: Supplementary file 7 — Fig S7 [file CNS-26-1303-s007.tif]

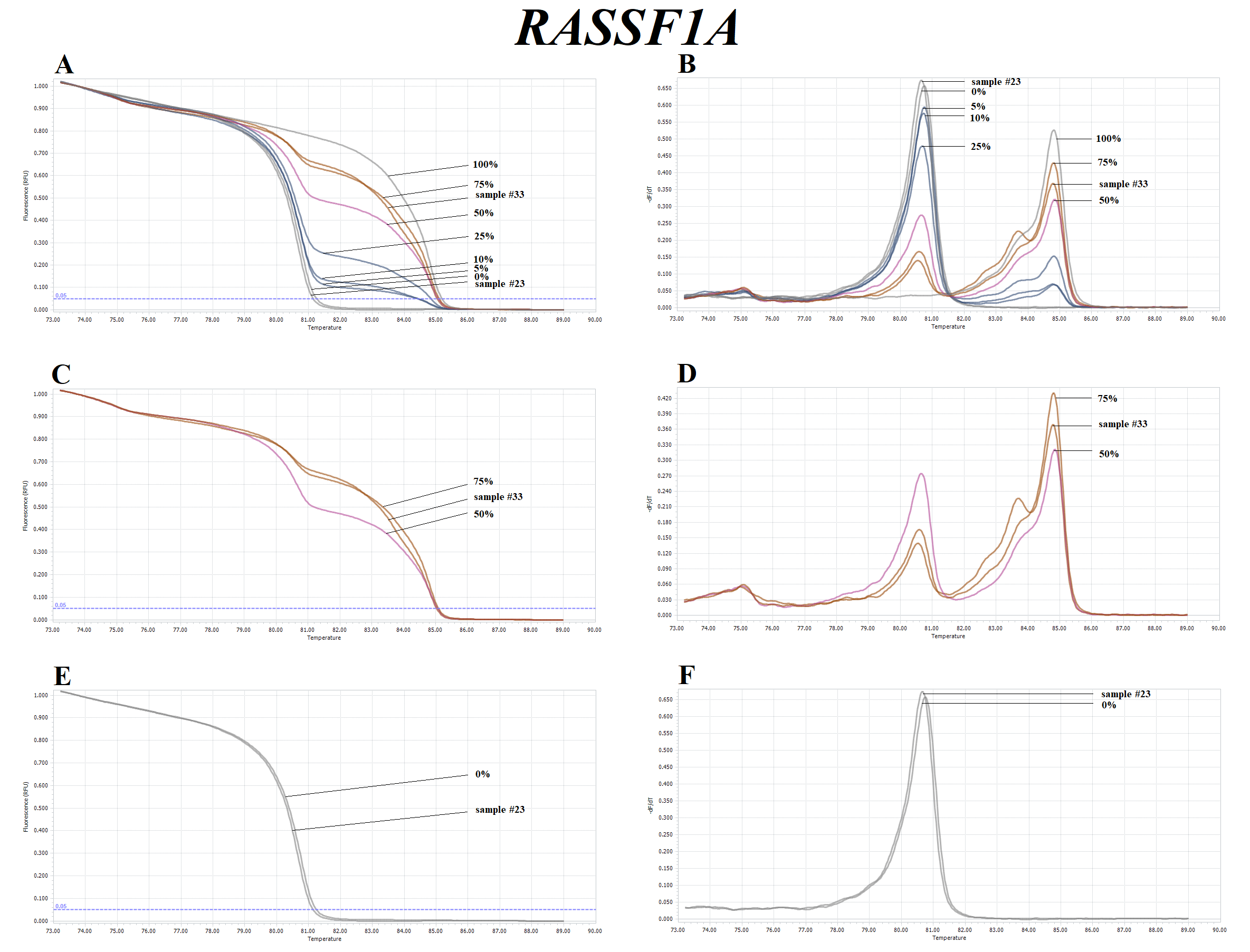

Supplement: Supplementary file 8 — Fig S8 [file CNS-26-1303-s008.tif]

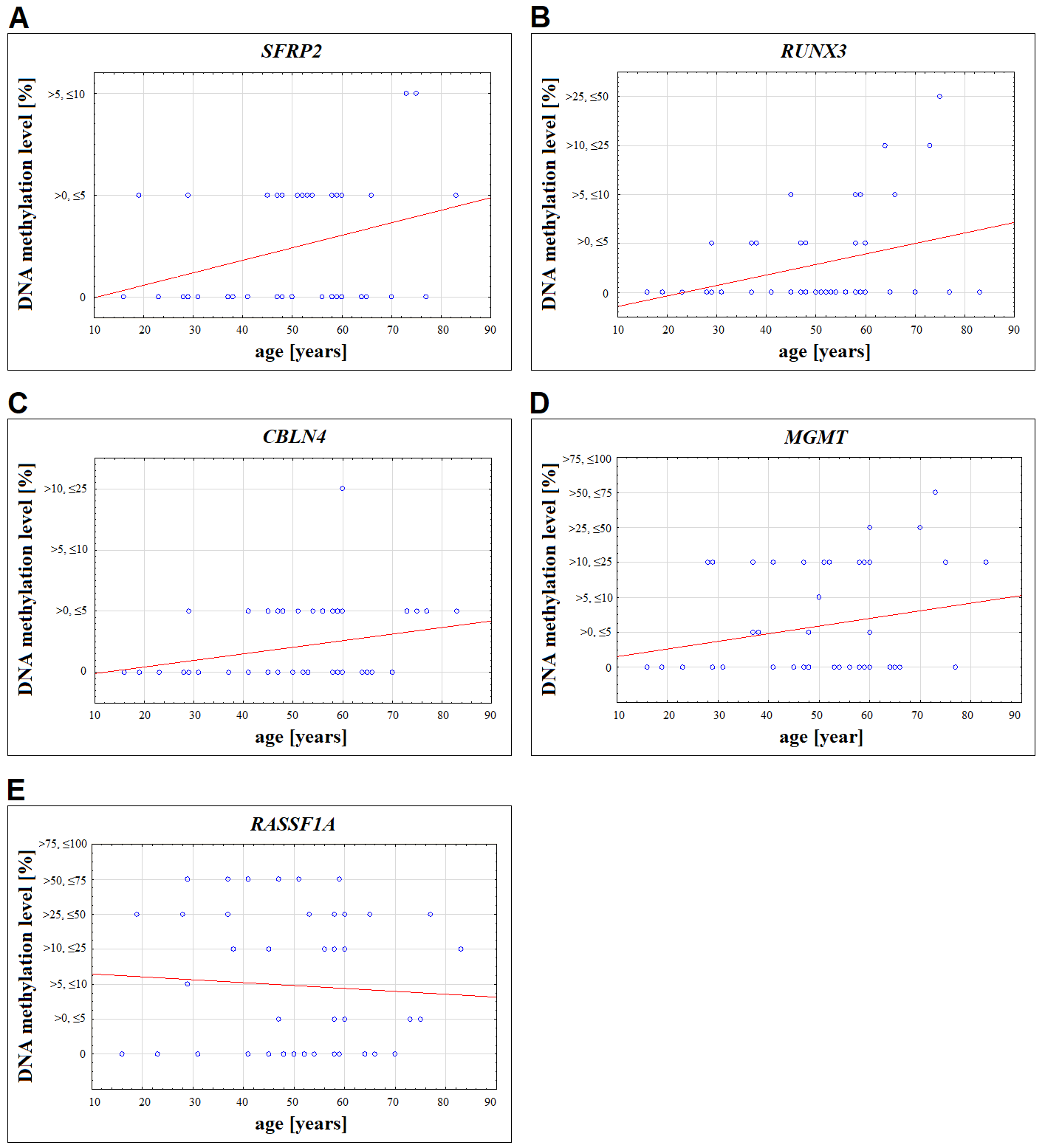

Supplement: Supplementary file 9 — Fig S9 [file CNS-26-1303-s009.tif]
