# Supplementary material for: DNA methylation analysis with methylation‐sensitive high‐resolution melting (MS‐HRM) reveals gene panel for glioma characteristics
Source: CNS Neurosci Ther. 2020 Aug 11;26(12):1303–14. doi: 10.1111/cns.13443 (PMC7702229; doi:10.1111/cns.13443)
Supplement: Supplementary file 10 — Fig S10 [file CNS-26-1303-s010.docx]

**Fig. S10**

*MGMT*

chr10:129,466,590-129,467,489

ATAGGTTTTGGAGGTTGTTTTTACGGTTTTTTGATAGGGTTTTTGTTGGTTTGGGGGTTT

TTGATTAGGGGAGCGGTATTAGGAGGGGAGAGATTCGCGTTTCGGGTTTAGCGTAGTCGT

TTCGAGTAGGATCGGGATTTTTATTAAGCGGGCGTCGTTTTACGATTTTCGCGCGTTTTT

AGGATTATTCGGGTACGTGGTAGGTCGTTTGTACGTTCGCGGATTATTTTTGTGATAGGA

AAAGGTACGGGTTATTTGGTAAATTAAGGTATAGAGTTTTAGGCGGAAGTTGGGAAGGCG

TCGTTCGGTTTGTATCGGTCGAAGGGTTATTCGGGTTAGGCGTATAGGGTAGCGGCGTTG

TCGGAGGATTAGGGTCGGCGTGTCGGCGTTTAGCGAGGATGCGTAGATTGTTTTAGGTTT

GGCGTCGTCGTATAGGGTATGCGTCGATTCGGTCGGGCGGGAATATTTCGTTTTTTTCGG

GTTTCGTTTTAGTTTCGTTTTCGCGCGTTTCGGTTTCGTTTTCGCGCGTTTTTTTGTTTT

TTTTAGGTTTTCGGTTTCGTTTCGTTTTAGATTTCGTTTTACGTCGTTATTTTCGTGTTT

**TTCGGTTTCGTTTTCGCGTTTCGGATATGTTGGGATAGTTCGCGTTTTTAGAACGTTTTG**

**CGTTTCGACGTTCGTAGGTTTTCGCGGTGCGTATCGTTTGCGATTTG**GTGAGTGTTTGGG

TCGTTTCGTTTTCGGAAGAGTGCGGAGTTTTTTTTCGGGACGGTGGTAGTTTCGAGTGGT

TTTGTAGGCGTTTTTATTTCGTCGTCGGGTGTGGGGTCGTTTTGATTTTTATTTATTTCG

GGCGAGTTTTAGGTGCGTTTTAAGTGTTTTTTAGGTGTTGTTTAGTTTTTTTTCGGGTTT

*MGMT* exon 1 is highlighted with red. CpG dinucleotides analyzed with pyrosequencing are highlighted with yellow and underlined. Sequence fragments corresponding to primers used for MS-HRM analysis are underlined and CpGs analyzed in this method are highlighted with blue.

A)

B)


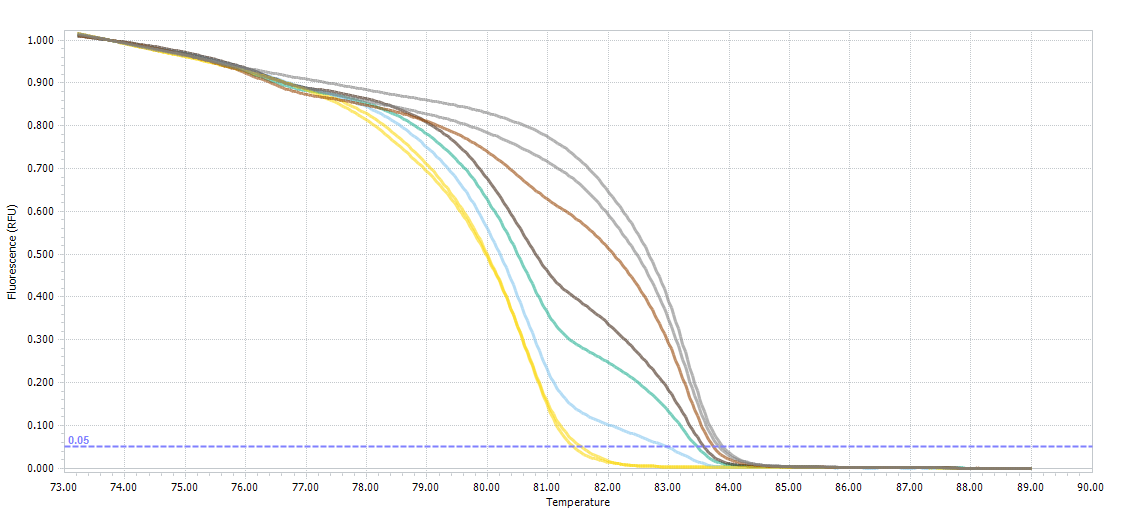


**Fig. S10a**

A) Pyrosequencing result of *MGMT* methylation analysis of sample #3. The y axis represents the signal intensity, while the x axis shows the dispensation order. The blue color indicate the % of methylation at each CpG site. B) MS-HRM result of this sample was also 0% (normalized melting curve of this sample is indicated with yellow and pointed with an arrow).

A)

B)


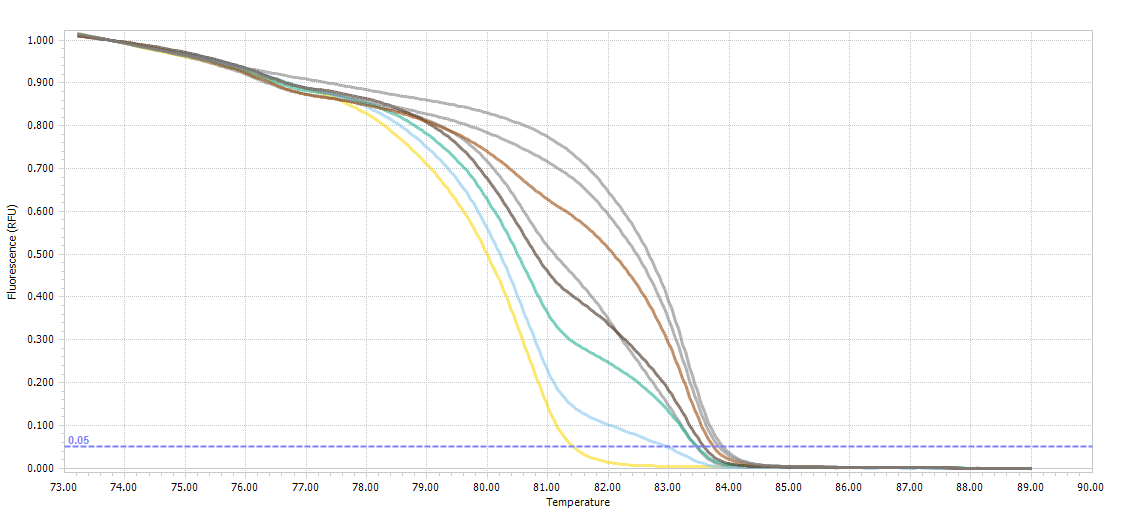


**Fig. S10b**

A) Pyrosequencing result of *MGMT* methylation analysis of sample #10 (average of all five CpGs = 29.2%). The y axis represents the signal intensity, while the x axis shows the dispensation order. The blue color indicate the % of methylation at each CpG site. B) For this sample MS-HRM result was in the range of >25, ≤50% (normalized melting curve of this sample is indicated with grey and pointed with an arrow).

A)

B)


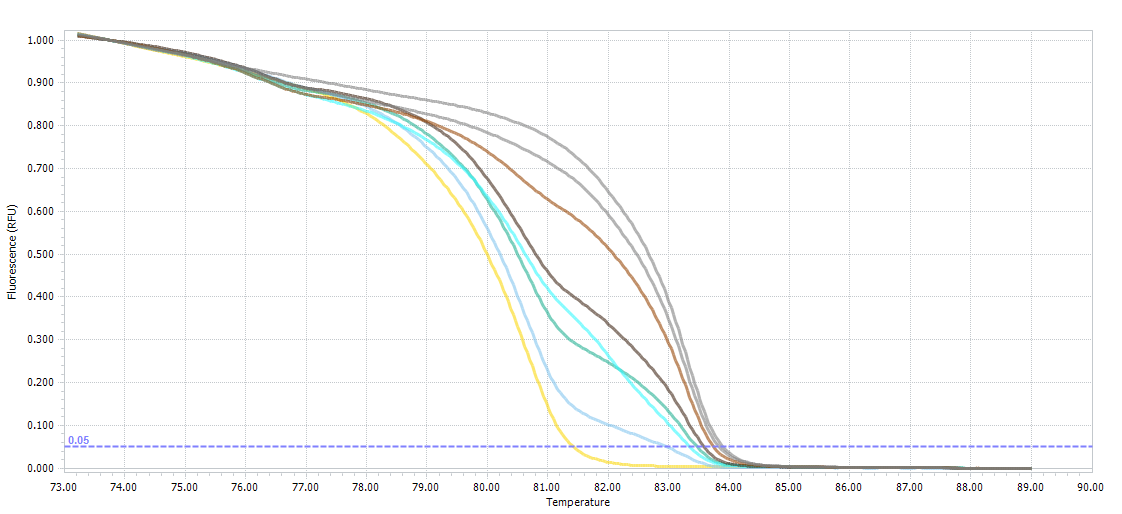


**Fig. S10c**

A) Pyrosequencing result of *MGMT* methylation analysis of sample # 12(average of all five CpGs = 24.6%). The y axis represents the signal intensity, while the x axis shows the dispensation order. The blue color indicate the % of methylation at each CpG site. B) For this sample MS-HRM result was in the range of >10, ≤25% (normalized melting curve of this sample is indicated with turquoise and pointed with an arrow).

A)

B)


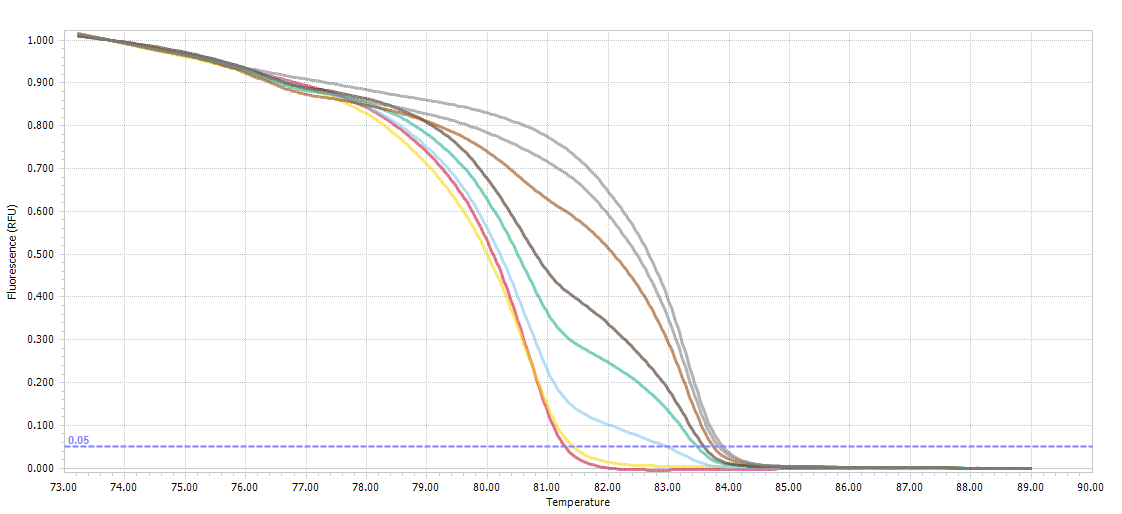


**Fig. S10d**

A) Pyrosequencing result of *MGMT* methylation analysis of sample #1 (average of all five CpGs = 3.2%). The y axis represents the signal intensity, while the x axis shows the dispensation order. The blue color indicate the % of methylation at each CpG site. B) For this sample MS-HRM result was 0% (normalized melting curve of this sample is indicated with red, and pointed with an arrow).

A)

A)

B)


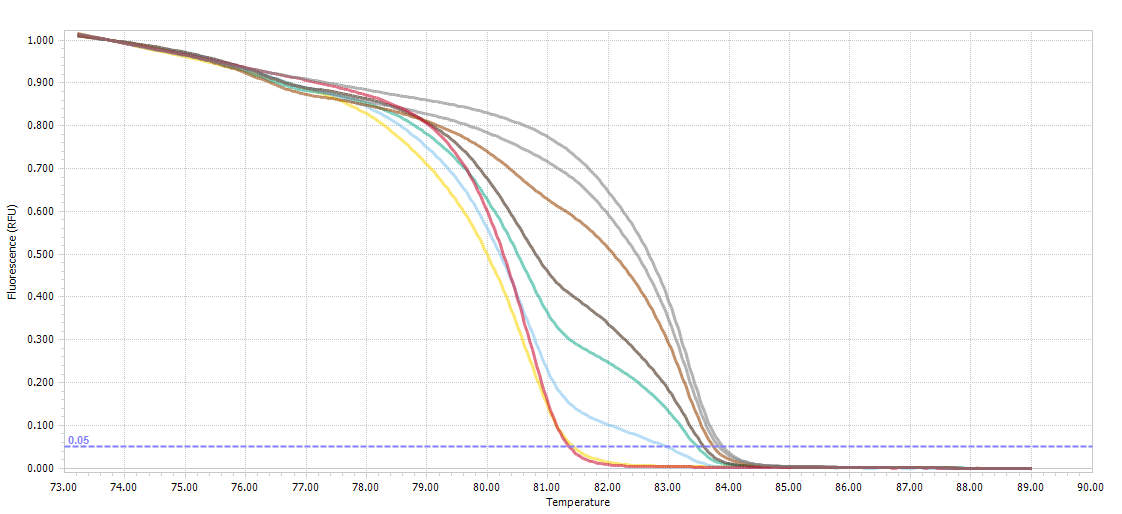


**Fig. S10e**

Figure. A) Pyrosequencing result of *MGMT* methylation analysis of sample #42 (average of all five CpGs = 8.6%). The y axis represents the signal intensity, while the x axis shows the dispensation order. The blue color indicate the % of methylation at each CpG site. B) For this sample MS-HRM result was in a range of 0-5% (normalized melting curve of this sample is indicated with red, and pointed with an arrow).

A)

B)


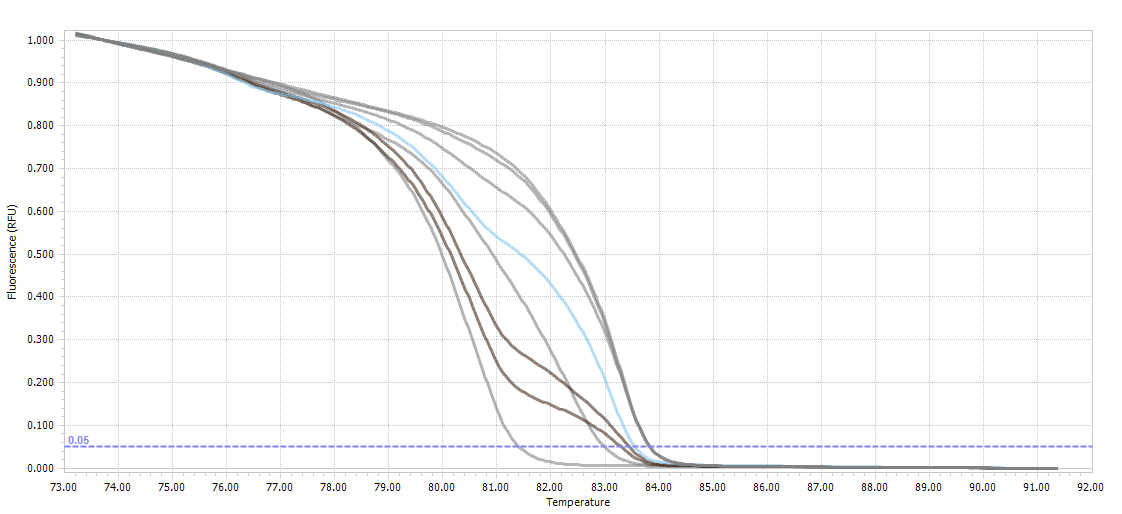


**Fig. S10f**

A) Pyrosequencing result of *MGMT* methylation analysis of sample #40 (average of all five CpGs = 21.4%). The y axis represents the signal intensity, while the x axis shows the dispensation order. The blue color indicate the % of methylation at each CpG site. B) For this sample MS-HRM result was in the range of >10, ≤25% (normalized melting curve of this sample is indicated with grey, and pointed with an arrow).

A)

B)


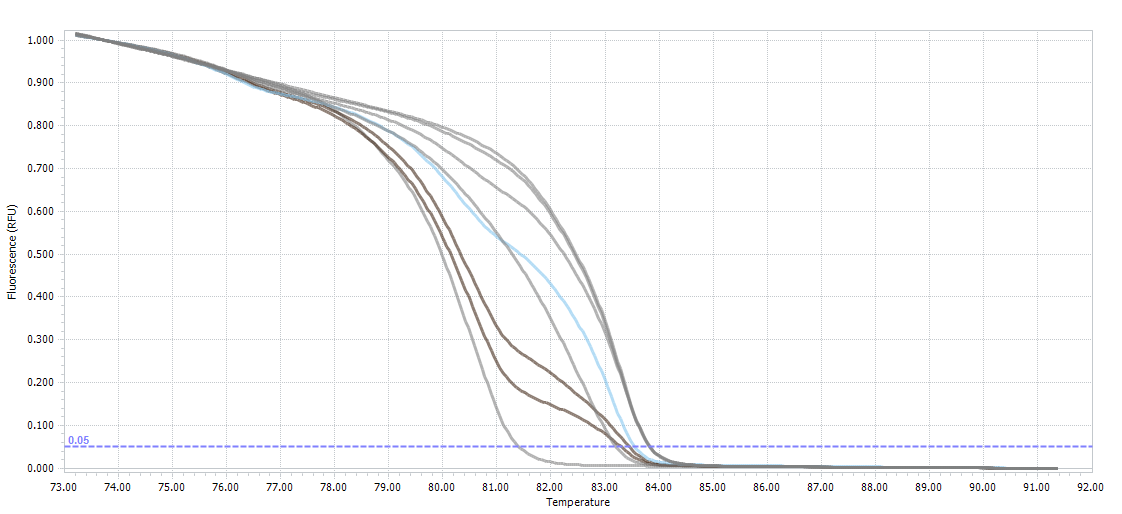


**Fig. S10g**

A) Pyrosequencing result of *MGMT* methylation analysis of sample #31 (average of all five CpGs = 48.8%). The y axis represents the signal intensity, while the x axis shows the dispensation order. The blue color indicate the % of methylation at each CpG site. B) For this sample MS-HRM result was in the range of >10, ≤25% (normalized melting curve of this sample is indicated with grey, and pointed with an arrow).

A)

B)


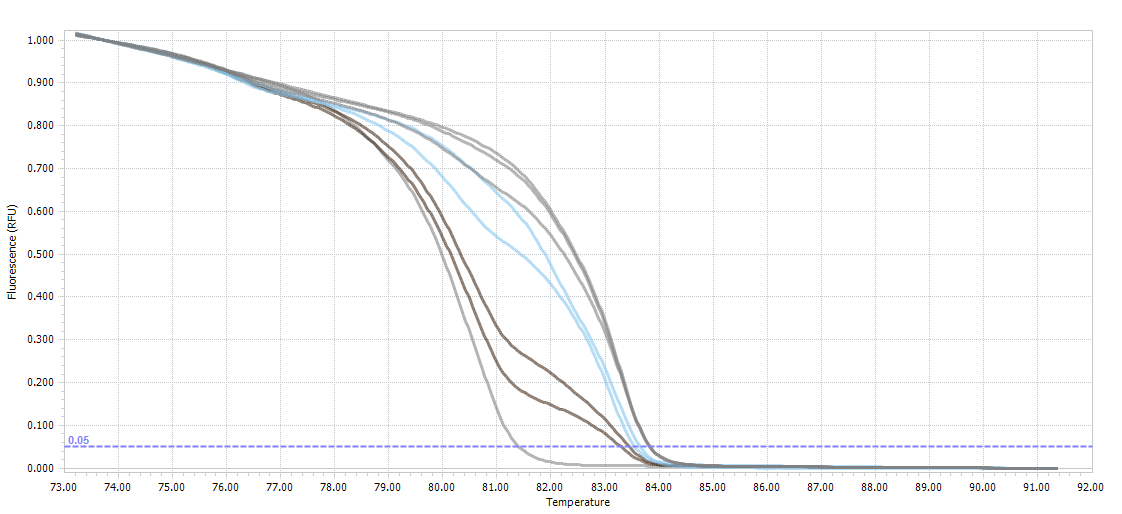


**Fig. S10h**

A) Pyrosequencing result of *MGMT* methylation analysis of sample #22 (average of all five CpGs = 73.4%). The y axis represents the signal intensity, while the x axis shows the dispensation order. The blue color indicate the % of methylation at each CpG site. B) For this sample MS-HRM result was in the range of >25, ≤50% (normalized melting curve of this sample is indicated with blue, and pointed with an arrow).

A)

B)


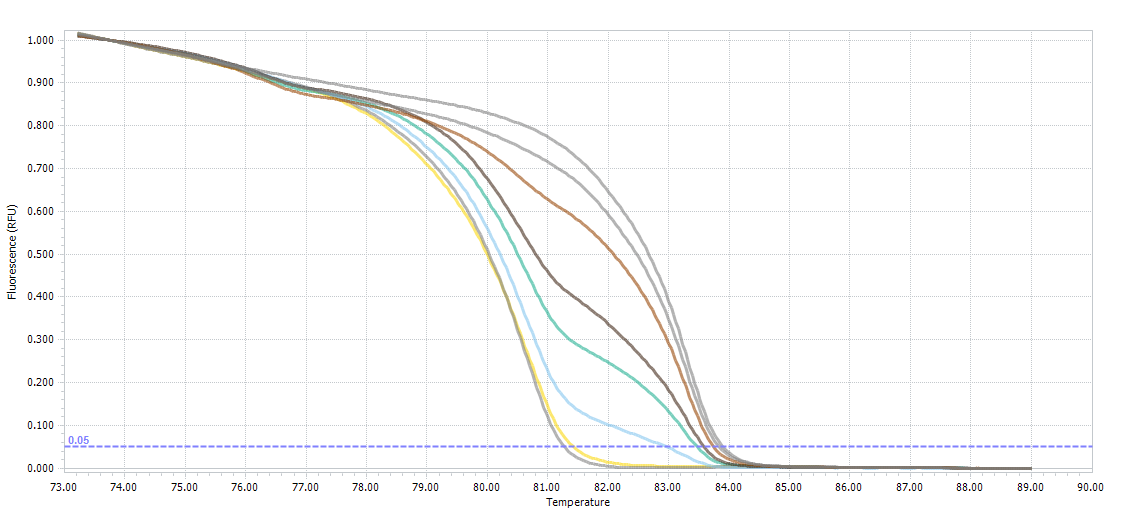


**Fig. S10i**

A) Pyrosequencing result of *MGMT* methylation analysis of sample #11 (average of all five CpGs = 3.8 %). The y axis represents the signal intensity, while the x axis shows the dispensation order. The blue color indicate the % of methylation at each CpG site. B) For this sample MS-HRM result was in 0% (normalized melting curve of this sample is indicated with grey, and pointed with an arrow).

A)

B)


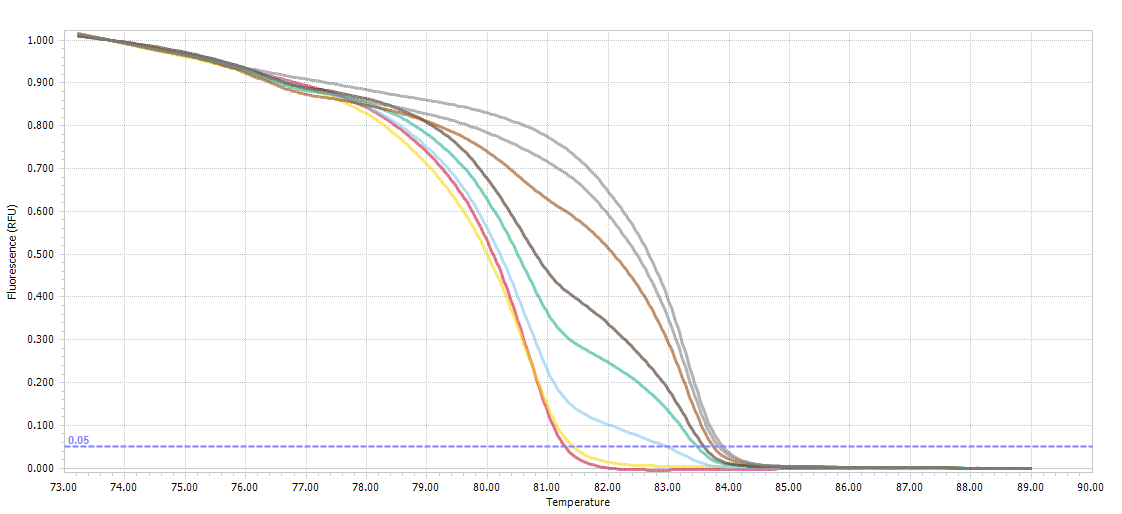


**Fig. S10j**

Figure. A) Pyrosequencing result of *MGMT* methylation analysis of sample #7 (average of all five CpGs = 3.6 %). The y axis represents the signal intensity, while the x axis shows the dispensation order. The blue color indicate the % of methylation at each CpG site. B) For this sample MS-HRM result was in 0% (normalized melting curve of this sample is indicated with red, and pointed with an arrow).
