# Supplementary material for: DNA methylation analysis with methylation‐sensitive high‐resolution melting (MS‐HRM) reveals gene panel for glioma characteristics
Source: CNS Neurosci Ther. 2020 Aug 11;26(12):1303–14. doi: 10.1111/cns.13443 (PMC7702229; doi:10.1111/cns.13443)
Supplement: Supplementary file 11 — Table S1 [file CNS-26-1303-s011.doc]

**Supplementary Table 1.**

The characteristics of the proposed 7-gene panel with promoter methylation significance in CNS tumors.

| **Gene** | **Encoded protein** | **Protein function** | **Promoter methylation /expression change** | **Effect of promoter methylation / expression change** | **Reference** |
| --- | --- | --- | --- | --- | --- |
| *SFRP1* | Secreted frizzled-related protein 1 | Wnt/β-catenin pathway antagonist | hypermethylation | high grade gliomas | Kafka et al. 2018 |
| *SFRP2* | Secreted frizzled-related protein 2 | Wnt/β-catenin pathway antagonist/agonist | hypermethylation | esophageal squamous cell carcinoma development | Wu et al. 2020  Liu et al. 2019 |
| *RUNX3* | Runt-related transcription factor 3 | Wnt/β-catenin pathway  antagonist | hypermethylation | WHO grade II meningiomas | Majchrzak-Celińska et al. 2015 |
| *CBLN4* | Cerebellin 4 precursor | *trans*-synaptic cell adhesion molecule, formation and maintenance of inhibitory GABAergic connections | knockout | reduction of GABAergic connections | Chacón et al. 2015  Seigneur and Südhof 2018 |
| *INA* | Internexin neuronal intermediate filament protein α | cellular matrix neurofilament | hypermethylation | short term GBM survivors | Shinawi et al. 2013 |
|  |  |  | expression | 1p/19q co-deleted tumors = good prognosis | Suh et al. 2013 |
| *MGMT* | O^6^-methylguanine-DNA methyl-transferase | removing alkyl adducts from the O^6^ position of guanine in DNA | hypermethylation | longer OS, PFS, better response to TMZ therapy | Binabaj et al. 2018  Stupp et al. 2009  Hegi et al. 2005 |
| *RASSF1A* | Ras association domain family 1 isoform A | tumor suppressor | hypermethylation | carcinogenesis | Malpeli et al. 2019 |
