# Supplementary material for: DNA methylation analysis with methylation‐sensitive high‐resolution melting (MS‐HRM) reveals gene panel for glioma characteristics
Source: CNS Neurosci Ther. 2020 Aug 11;26(12):1303–14. doi: 10.1111/cns.13443 (PMC7702229; doi:10.1111/cns.13443)
Supplement: Supplementary file 12 — Table S2 [file CNS-26-1303-s012.doc]

Supplementary Table 2. PCR primer sequences

| **Gene** | **Primer sequence** | | **T_A_ [˚C]** | **Screened CpGs/Product size [bp]** | **References** |
| --- | --- | --- | --- | --- | --- |
| *SFRP1* | F | 5`AAGTTTTAAGGTAAGGTTGTTTTT3` | 60 | 12/132 | Amornpisutt et al. 2015 |
|  | R | 5`AACCCTCAATCCCCAACAC3` |  |  |  |
| *SFRP2* | F | 5`TTTCGGATTGGGGTAAAATAAGT3` | 60 | 20/154 | Xiao et al. 2014 |
|  | R | 5`CGCTACGCCCCCACAATA3` |  |  |  |
| *RUNX3* | F | 5`TTTTTAGAGAATGAGGGATTTTTGT3` | 55 | 7/115 | Li et al. 2014 |
|  | R | 5`CCCTAATCCCTTAAATCTAATACCC3` |  |  |  |
| *INA* | F | 5`GTTCGTTTGGATTTGGAGA3` | 61 | 8/93 | Designed using Methyl Primer Express v 1.0 software (Applied Biosystems) |
|  | R | 5`CAACAACTCGACTACCTCCTC3` |  |  |  |
| *CBLN4* | F | 5`GCGAGTTTAGGGAAGTTTTG3` | 60 | 7/107 | Designed using Methyl Primer Express v 1.0 software (Applied Biosystems) |
|  | R | 5`CTACGACTTCTAAAACTCATCTTCC3` |  |  |  |
| *MGMT* | F | 5`CGTTTGCGATTTGGTGAGTGTT3` | 60 | 5/94 | Wojdacz et al. 2007 |
|  | R | 5`CCTACAAAACCACTCGAAACTACCA3` |  |  |  |
| *RASSF1A* | F | 5`AGTTTGGATTTTGGGGGAGG3` | 60 | 12/136 | Stuopelytė et al. 2013 |
|  | R | 5`CAACTCAATAAACTCAAACTCCCC3` |  |  |  |

F- forward primer, R- reverse primer, T_A_ – annealing temperature
