# Supplementary material for: DNA methylation analysis with methylation‐sensitive high‐resolution melting (MS‐HRM) reveals gene panel for glioma characteristics
Source: CNS Neurosci Ther. 2020 Aug 11;26(12):1303–14. doi: 10.1111/cns.13443 (PMC7702229; doi:10.1111/cns.13443)
Supplement: Supplementary file 13 — Table S3 [file CNS-26-1303-s013.docx]

**Supplementary Table 3**

The relationship between gene promoter methylation level and patients overall survival time. No statistically significant difference (p < 0.05) was observed.

| Gene | Number of analyzed cases | Overal survival time - Median [months] | Overall survival time - Min [months] | Overall survival time - Max [months] | R | p |
| --- | --- | --- | --- | --- | --- | --- |
| *SFRP1* | 27 | 10 | 3 | 42 | -0,0241 | 0,9049 |
| *SFRP2* | 26 |  |  |  | -0,2005 | 0,3262 |
| *RUNX3* | 27 |  |  |  | -0,0453 | 0,8223 |
| *CBLN4* | 26 |  |  |  | 0,0942 | 0,6472 |
| *INA* | 26 |  |  |  | -0,1465 | 0,475 |
| *MGMT* | 27 |  |  |  | 0,195 | 0,3298 |
| *RASSF1A* | 27 |  |  |  | 0,1801 | 0,3688 |

Min – shortest overall survival time in the analyzed group of patients, Max – longest overall survival time in the analyzed group of patients, R – Spearman rank correlation coefficient,
